# The molecular tweezer CLR01 improves behavioral deficits and reduces tau pathology in P301S-tau transgenic mice

**DOI:** 10.1186/s13195-020-00743-x

**Published:** 2021-01-04

**Authors:** Jing Di, Ibrar Siddique, Zizheng Li, Ghattas Malki, Simon Hornung, Suman Dutta, Ian Hurst, Ella Ishaaya, Austin Wang, Sally Tu, Ani Boghos, Ida Ericsson, Frank-Gerrit Klärner, Thomas Schrader, Gal Bitan

**Affiliations:** 1 Department of Neurology, David Geffen School of Medicine, University of California, Gordon Neuroscience Research Building, Room 451, 635 Charles E. Young Drive South, Los Angeles, CA 90095-7334 USA; 2 Present Address: Division of Peptide Biochemistry, Technical University of Munich, Freising, Germany; 3 Faculty of Chemistry, University of Duisburg-Essen, Essen, Germany; 4 Brain Research Institute, University of California, Los Angeles, CA USA; 5 Molecular Biology Institute, University of California, Los Angeles, CA USA

**Keywords:** Alzheimer’s disease, Tauopathy, Small molecule, Mouse model, Immunohistochemistry, Seeding

## Abstract

**Background:**

Molecular tweezers (MTs) are broad-spectrum inhibitors of abnormal protein aggregation. A lead MT, called CLR01, has been demonstrated to inhibit the aggregation and toxicity of multiple amyloidogenic proteins in vitro and in vivo. Previously*,* we evaluated the effect of CLR01 in the 3 × Tg mouse model of Alzheimer’s disease, which overexpresses mutant human presenilin 1, amyloid β-protein precursor, and tau and found that subcutaneous administration of the compound for 1 month led to a robust reduction of amyloid plaques, neurofibrillary tangles, and microgliosis. CLR01 also has been demonstrated to inhibit tau aggregation in vitro and tau seeding in cell culture, yet because in Alzheimer’s disease (AD) and in the 3 × Tg model, tau hyperphosphorylation and aggregation are thought to be downstream of Aβ insults, the study in this model left open the question whether CLR01 affected tau in vivo directly or indirectly.

**Methods:**

To determine if CLR01 could ameliorate tau pathology directly in vivo, we tested the compound similarly using the P301S-tau (line PS19) mouse model. Mice were administered 0.3 or 1.0 mg/kg per day CLR01 and tested for muscle strength and behavioral deficits, including anxiety- and disinhibition-like behavior. Their brains then were analyzed by immunohistochemical and biochemical assays for pathological forms of tau, neurodegeneration, and glial pathology.

**Results:**

CLR01 treatment ameliorated muscle-strength deterioration, anxiety-, and disinhibition-like behavior. Improved phenotype was associated with decreased levels of pathologic tau forms, suggesting that CLR01 exerts a direct effect on tau in vivo. Limitations of the study included a relatively short treatment period of the mice at an age in which full pathology is not yet developed. In addition, high variability in this model lowered the statistical significance of the findings of some outcome measures.

**Conclusions:**

The findings suggest that CLR01 is a particularly attractive candidate for the treatment of AD because it targets simultaneously the two major pathogenic proteins instigating and propagating the disease, amyloid β-protein (Aβ), and tau, respectively. In addition, our study suggests that CLR01 can be used for the treatment of other tauopathies in the absence of amyloid pathology.

**Supplementary information:**

The online version contains supplementary material available at 10.1186/s13195-020-00743-x.

## Background

Tau is a microtubule-associated protein, important in the assembly and stabilization of microtubules. Tau is a natively unstructured protein with little intrinsic tendency to aggregate in vitro, yet a class of neurodegenerative disorders called tauopathies, including Alzheimer’s disease (AD), progressive supranuclear palsy (PSP), corticobasal syndrome (CBS), argyrophilic grain disease (AGD), Pick's disease (PiD), and frontotemporal dementia (FTD) [1], is characterized by aggregation of aberrantly post-translationally modified tau in the brain. Aggregation of tau in tauopathies leads to formation of paired helical filaments, neurofibrillary tangles (NFTs), and other types of fibrillar deposits, which are the pathological hallmarks of these diseases [1].

Humans have six isoforms of tau formed by alternative splicing of exons 2, 3, and 10 in the cognate gene, MAPT. These isoforms are designated by the number of N-terminal exons (0, 1, or 2) and the number of repeats, 3 or 4, in the microtubule-binding domain (for example, the longest isoform is 2N4R) [2]. In tauopathies, abnormal post-translational modifications, primarily hyperphosphorylation, of tau lead to self-assembly of the protein into toxic oligomers and aggregates that cause neuronal dysfunction and death [3, 4]. Aggregation of hyperphosphorylated tau is thought to occur in a nucleation-dependent manner and seeding of the aggregation by oligomers or fibril fragments in naïve recipient cells in a prion-like manner is a major mechanism by which tauopathies are thought to progress throughout the brain [5–7].

In AD, the most common tauopathy, amyloid plaques comprising predominantly the 42-amino acid residues form of amyloid β-protein (Aβ42) and neurofibrillary tangles made of hyperphosphorylated tau are pathological hallmarks of the disease. Aβ oligomers are thought to initiate the disease process in AD [8], whereas tau pathology is believed to be the main propagator of pathology through the brain [9]. Thus, NFT burden, but not plaque abundance, correlates with the degree of cognitive impairment in AD [10, 11]. Interestingly, Aβ oligomers are thought to facilitate cell-to-cell propagation of tau [12, 13] in a mechanism that is unique to AD among all the tauopathies and may explain why AD is highly prevalent whereas other tauopathies are rare [14]. These considerations may explain, at least partially, the reason for the repeated failure of AD therapies targeting only Aβ or only tau pathologies and suggest that targeting the self-assembly, toxicity, and brain propagation of both proteins may be necessary for successful therapy.

**Fig. 1:**
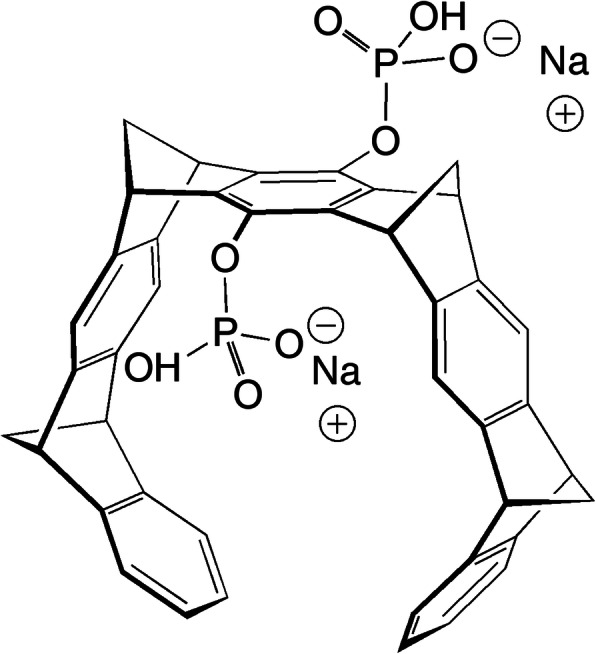
Schematic 3-dimensional structure of CLR01. The compound is partially protonated at pH 7.4

Molecular tweezers (MTs) are small molecules acting as broad-spectrum modulators/inhibitors of abnormal protein self-assembly [15–17], including the oligomerization and aggregation of Aβ and tau. Initial studies largely have been dedicated to the effect of the lead MT, CLR01 (Fig. 1), on Aβ. CLR01 was shown to inhibit the aggregation of both Aβ40 and Aβ42 [18] and their toxicity in cell lines and primary cultures [18–20]. We also demonstrated that CLR01 inhibited Aβ oligomerization [18, 21] and protected membranes from disruption by Aβ42 [22]. In vivo, CLR01 was evaluated in the triple-transgenic (3 × Tg) mouse model of AD, which overexpresses mutant forms of human presenilin 1 (PS1), amyloid β-protein precursor (APP), and tau [23]. The mice were treated for 28 days with 0.04 mg/kg per day CLR01 or vehicle (sterile saline) administered subcutaneously (s.c.), continuously using osmotic minipumps. The treatment resulted in a significant decrease in amyloid plaques, neurofibrillary tangles, and microgliosis [20]. Similarly, in a small study using a Tg rat model expressing familial AD-linked mutant forms of human APP and PS1 [24], administration of 0.1 or 0.3 mg/kg per day CLR01 in a similar manner led to 45% and 52% reduction in plaque burden, respectively [25].

Initial in vitro experiments testing the effect of CLR01 on tau showed that the compound inhibited completely at a 1:1 concentration ratio the aggregation of the shortest form of tau, 0N3R, induced to aggregate by arachidonic acid [18]. More recently, CLR01 was found to inhibit the aggregation of the longest form of tau, 2N4R, either when it was induced to aggregate by heparin or when the protein was phosphorylated in vitro rendering it prone to aggregation [26]. CLR01 also inhibited seeded, intracellular tau aggregation in a biosensor cell line [26]. The main binding region for CLR01 on tau was identified by NMR [26] and mass-spectrometry [27] to be the repeat domain, which forms the core of the aggregates.

Although upon treatment with CLR01 NFTs were reduced in the 3 × Tg mice, the model did not allow distinguishing between a direct effect of CLR01 on tau, which likely would necessitate internalization of the compound into the affected neurons, and an indirect effect via reduction of Aβ pathology. To differentiate between these two possibilities, here, we tested the effect of CLR01 in a mouse model of tauopathy that lacks Aβ pathology, the P301S-tau (PS19 line) mouse [28].

## Materials and methods

### Experimental drug preparation

CLR01 was synthesized and purified as a sodium salt as described previously [29]. The compound was prepared as a 10-mM stock solution in sterile deionized water and diluted to the required concentrations in 0.9% (w/v) sterile saline.

### Experimental model of tauopathy

P301S-tau (Tg(Prnp-MAPT*P301S)PS19Vle) transgenic mice were obtained from Jackson laboratories and were bred and maintained by the UCLA Division of Laboratory Animal Medicine. Homozygous P301S-tau and wild-type littermates were used in this study. Mice were housed in groups of 5, under a 12-h light/12-h dark cycle at 21 °C, with food and water ad libitum*.* All the experiments were reviewed and carried out in accordance with National Research Council Guide for the Care and Use of Laboratory Animals, approved by the University of California-Los Angeles Institutional Animal Care Use Committee, and performed with strict adherence to the guidelines set out in the National Institutes of Health Guide for the Care and Use of Laboratory Animals.

### Experimental drug administration

CLR01 at 0.3 or 1.0 mg/kg per day or vehicle (0.9% sterile saline) was administered subcutaneously using osmotic minipumps (Alzet Model 1004), which release the solution at 0.11 μL/h. The pumps were implanted as described previously [20]. The mice were randomized into five treatment groups, each containing 8 ± 1 females and 8 ± 1 males. Treatment groups of transgenic, P301S-tau mice received vehicle, 0.3, or 1.0 mg/kg per day CLR01. Control, wild-type littermate groups received vehicle or 1.0 mg/kg CLR01 per day. The treatment started when animals were 26–28 weeks of age and continued for 35 days. Animals were monitored daily for changes in behavior or appearance. Bodyweight was measured on days 1, 17/18, and 35 of the treatment. At the end of the treatment, the animals were sacrificed, their blood was collected for serum preparation, and their brain was extracted for biochemical and immunohistochemical analyses.

### Grip strength test

The neuromuscular function of the animals was measured using a grip-strength test [30] on day 1, before pump implantation, on day 17 or 18 of the treatment, and on day 35, before sacrificing the mice. Briefly, a 43 × 43-cm wire mesh made of 1-mm diameter wire and consisting of 12 × 12-mm squares was used as an inverted screen. Mice were placed at the center of the screen, which then was rotated to an inverted position. The screen was placed over the open-field box, 40-cm above a padded surface for a maximum of 180 s. The latency of the mouse to fall was recorded or noted to be 180 s if the mouse did not fall. The test was repeated four times per animal with 60 s intervals between trials.

### Open-field test

Activity, anxiety-like behavior, and exploration pattern were monitored in an open-field arena. The test was performed on day 1, before pump implantation, and on day 35, before sacrificing the mice. Each mouse was placed at the center of a 40 × 40-cm box and allowed to explore freely for 5 min. The box was cleaned with 70% ethanol between animals. The tests were video-recorded using ANY-maze (Wood Dale, IL). For the analysis, the box was divided into outer and inner zones, the latter consisting of a 22.5 × 22.5-cm square in the center of the field. Parameters including average duration in the center, latency to first center entry, number of entries into the center, ratio of time spent in the periphery versus the center, average speed, and total distance traveled were measured and calculated using the ANY-maze program. In addition, the number and time of freezing episodes and the number and time of rearing events were analyzed manually by an experimenter blinded to genotype and treatment.

### Euthanasia and tissue processing

Mice were anesthetized using isoflurane and perfused transcardially using 0.1 M phosphate-buffered saline (PBS), pH 7.4, containing 1% protease and phosphatase inhibitors (PPI, Sigma-Aldrich). Brains were removed and divided into the two hemispheres. One hemisphere was fixed immediately by immersion in 4% (w/v) paraformaldehyde in 0.1 M PBS for immunohistochemistry. The other hemisphere was dissected into four regions of interest, including cortex, hippocampus, cerebellum, and midbrain + brainstem, snap-frozen, and stored at − 80 °C for biochemical and seeding analyses. These brain regions were homogenized mechanically while frozen (in the presence of liquid nitrogen) using a CryoGrinder (OPS Diagnostics, Lebanon, NJ). The mortar and pestle were cleaned with 70% ethanol after every sample and placed in liquid nitrogen to cool for 10 min before use. The resulting sample powder was stored at − 80 °C.

The powder from each sample was suspended in extraction buffer comprising Tris-buffered saline (TBS), pH 7.4, supplemented with PPI, benzonase (1 unit/μL), and 2 mM MgCl_2_. The samples were vortexed and incubated at room temperature for 30 min with shaking, centrifuged at 20,000*g* for 30 min at 4 °C, and the soluble-fraction supernates were transferred to new tubes and stored at − 80 °C. The pellets were resuspended in extraction buffer supplemented with 1% n-dodecyl-β-D-maltoside, vortexed briefly, and incubated for 30 min at room temperature with shaking. The samples then were centrifuged at 100,000*g* for 15 min at 4 °C and the membrane-bound-fraction supernates were transferred to new tubes and stored at − 80 °C. The pellets were suspended in TBS containing 5 M Gu∙HCl and sonicated for 10 min. These suspensions were labeled insoluble fraction and stored at − 80 °C. Protein concentration was measured using a bicinchoninic acid (BCA) assay (ThermoFisher, Carlsbad, CA) in each fraction of each sample.

### Measurement of total tau in CNS-derived exosomes

Blood was obtained upon sacrificing the mice by a cardiac blood draw. The blood was allowed to clot for 10 min and then centrifuged at 1500*g* for 10 min at 4 °C. Clear sera were collected and stored at − 80 °C until further use. Frozen serum samples were thawed on ice. PPI were added immediately, followed by ExoQuick Exosome Precipitation Solution (System Biosciences). The samples were mixed gently and incubated on ice for 60 min and then centrifuged at 1500*g* for 30 min at 4 °C. The supernates were discarded, and the resulting pellets were suspended in chilled PBS containing 1% (w/v) bovine serum albumin (BSA) for the subsequent enrichment step. Two micrograms of anti-L1 neuronal cell-adhesion molecule antibody (clone 5G3, Santa Cruz Biotechnology) were used to coat 1 mg of M-270 epoxy Dynabeads using a Dynabeads Antibody Coupling Kit (Life Technologies) overnight at 37 °C with gentle rotation following the manufacturer’s instructions. Antibody-coated beads then were mixed gently with the isolated serum exosomes in cold PBS, pH 7.4, containing 1% (w/v) BSA, and incubated overnight at 4 °C with gentle rotation. The bead-attached exosomes were washed with 500 μL of 0.1% (w/v) BSA in PBS, pH 7.4, containing PPI and transferred into new tubes, in which the exosomes were lysed by incubating in 20 μL of radioimmunoprecipitation assay (RIPA) buffer (Thermo Fisher Scientific) containing PPI for 30 min at room temperature. The samples then were centrifuged at 12,000*g* for 10 min at 4 °C. The supernates containing enriched neuronal exosomal proteins were transferred to new tubes and stored at − 80 °C until further use. Ten micrograms of total neuronal exosomal proteins, as determined by a BCA assay, were used for each assay to determine total tau concentration using a Human Tau (Total) ELISA Kit (Invitrogen, Camarillo, CA).

### Brain histology and immunohistochemistry

The fixed hemispheres were sectioned coronally into 40-μm thick sections using a Leica VT1000 vibratome (Leica Biosystems). Sections were collected into multi-well plates containing 0.1 M PBS and 0.1% (w/v) sodium azide at 4 °C. Gallyas silver staining was performed using the FD NeuroSilver Kit II (PK 301/301A, Version 2014-01) following the manufacturer’s instructions. For immunofluorescence, PBS containing 0.3% (v/v) Triton X-100 (PBST, Sigma) was used in all washing steps. Free-floating sections were placed in wells of 24-well plates, rinsed for 10 min in PBST, and blocked for 60 min using a blocking buffer (BB) comprising 10% goat serum and 2% bovine serum albumin in 0.1 M PBS. Slices then were incubated with primary antibodies diluted in BB (Table 1) overnight at 4 °C with gentle agitation. Then, slices were washed thrice in Tris-buffered saline containing 0.1% (v/v) Tween-20 (TBS-T) for 10 min and incubated for 2 h at room temperature in the dark with secondary antibodies diluted in BB (Table 1). Slices again were washed thrice for 10 min and mounted onto gelatin-coated slides using ProLong™ Diamond Antifade mountant containing 4′,6-diamidino-2-phenylindole (DAPI, Invitrogen-Thermo Fisher). Slices were visualized using a Keyence BZ-X710 fluorescence microscope. All stereological cell counts and other analyses were performed by operators blinded to the treatment.

**Table 1:** Antibodies used in IHC

Target	Antibody/antigen	Dilution	Source	Secondary antibody	Dilution	Source
p-Tau	AT8 (pSer202, pThr205)	1:200	ThermoFisher	AlexaFluor555 (anti-mouse)	1:200	ThermoFisher
Microglia	Iba1	1:100	Abcam	AlexaFluor647 (anti-rabbit)	1:200	ThermoFisher
Astrocytes	GFAP	1:200	Cell Signaling	AlexaFluor594 (anti-mouse)	1:200	ThermoFisher
Neuronal Nuclei	NeuN	1:100	Cell Signaling	AlexaFluor647 (anti-rabbit)	1:200	ThermoFisher
Postsynapse	Homer	1:2000	Synaptic System	AlexaFluor594 (anti-guinea pig)	1:500	Synaptic System
Presynapse	Bassoon	1:200	Synaptic System	AlexaFluor647 (anti-rabbit)	1:500	ThermoFisher

### Enzyme-linked immunosorbent assay (ELISA)

The levels of total tau and tau phosphorylated at Thr231 (pT231-tau) in all three fractions of the transgenic mouse brain lysates were quantified using Invitrogen ELISA kits KHB0041 and KHB8051, respectively, according to the manufacturer’s instructions.

### Dot-blots

Nitrocellulose membranes were divided into grids using a pencil. Each grid was loaded with a sample of 3 μg total protein lysate from the soluble fraction in 10 μL. Membranes were dried and blocked using a blocking solution comprising 5% skim dried milk in TBS-T for 1 h at room temperature. Membranes then were incubated with monoclonal antibody TOC-1, diluted 1:5000 in blocking solution overnight at 4 °C. The membranes were washed thrice in TBS-T and incubated with HRP-conjugated goat anti-mouse secondary antibody (Santa Cruz Biotechnology) diluted 1:10,000 in blocking solution for 1 h at room temperature. Dots were visualized by incubating the membranes with ECL substrate (SuperSignal, West Pico PLUS, ThermoFisher) for 5 min and imaged using an Azure 300 Chemiluminescent Western Blot Imaging System (Azure biosystems).

### Native PAGE and western blots

Soluble fractions were thawed on ice and mixed with 4× NativePAGE sample buffer comprising 50 mM Bis-Tris, 50 mM NaCl, 10% (w/v) glycerol, and 0.001% Ponceau S, pH 7.2. Ten-microgram samples per well were loaded onto NativePAGE Novex 3–12% BisTris gels (ThermoFisher). The proteins were fractionated for 90–115 min at 150 V using an XCell *SureLock* Mini-Cell system (ThermoFisher, Carlsbad, CA). The fractionated proteins then were transferred to PVDF membranes for 1 h at 25 V using the XCell II™ Blot Module (ThermoFisher). After the transfer, the membranes were incubated in 20 mL of 8% acetic acid for 15 min to fix the proteins. The membranes were probed with anti-human tau antibody HT7 (ThermoFisher) or anti-phospho-tau antibody AT8 (ThermoFisher). The intensity of the protein bands was quantified densitometrically using Image J [31].

### Biosensor-cell seeding assay

The soluble and insoluble fractions were used as tau seeds. Membrane-bound fractions were not used because the detergent causes cytotoxicity. The measurements were performed as described previously [26]. Transduction complexes were made by combining 8.75 μL Opti-MEM (Gibco) and 1.25 μL Lipofectamine 2000 (Invitrogen) with 1 μg total protein of brain extracts diluted in Opti-MEM for a total volume of 20 μL per well and incubated at room temperature for 20 min before adding to the cells. The cells then were incubated with the transduction complexes for 48 h and subsequently visualized using fluorescence microscopy. The integrated FRET density was analyzed by flow cytometry as described previously [26, 32].

### Statistical analysis

We used Student’s *t* tests for comparison of two data sets, one-way analysis of variance (ANOVA) for comparison of more than two data sets, and two-way ANOVA for comparison of treatment groups across time or sex. In cases in which some data were missing, mixed models were used to compare data sets. Prism 8.0 (GraphPad, La Jolla, CA) was used for all the data analysis.

## Results

### Mouse model

A mutation in the MAPT gene that causes a P301S substitution in tau results in the early onset of familial frontotemporal dementia with parkinsonism linked to chromosome 17 [33, 34]. The PS19 line expresses human P301S-tau under the mouse prion protein (Prnp) promoter and shows progressively decreased synaptophysin immunoreactivity accompanied by microgliosis in the hippocampus starting at 3 months of age, followed by reduced LTP in the hippocampal CA1 region at 6 months of age. At this age, the mice develop NFT-like inclusions in the brainstem and spinal cord, whereas neuronal loss typically starts at 9 months of age [28]. Behaviorally, PS19 mice display progressive, age-associated motor deficits, including clasping and limb retraction when lifted by the tail, and cognitive deficits including increased hyperactivity and decreased anxiety-like behavior (disinhibition) [35]. As our study was designed to address particularly the question whether CLR01 could affect tau pathology in vivo in the absence of Aβ, we made the conservative choice to use relatively young mice, 6–6.5 months old, reasoning that the phenotype at this age would be sufficient for answering this specific question.

### Experimental design and safety assessment of CLR01 treatment

Previously, CLR01 was used to treat different animal models of proteinopathy using intracerebroventribular [32, 36] or subcutaneous administration. In the latter route, the compound was administered either via osmotic minipumps [20, 25, 36, 37], as was done here, or by daily injection, 2–7 days a week [38–41]. Here, we chose to use the less labor-intensive osmotic-minipump subcutaneous administration to answer the specific question whether CLR01 treatment affected tau directly.

In all of the previous studies, in which CLR01 was administered at doses ranging from 0.04 to 6.0 mg/kg per day, the treatment was found to be safe, and treatment-related adverse effects were not observed. Moreover, safety studies in wild-type mice showed that when administered as a single intraperitoneal injection at 100 mg/kg, CLR01 caused liver injury but did not affect other organs and did not reach lethal levels [42]. Chronic administration of the compound to wild-type mice for 30 days at 10 mg/kg caused a ~ 40% reduction in blood cholesterol as the only statistically meaningful finding [42], suggesting the CLR01 had a high safety margin. Nonetheless, because safety and toxicity may vary in transgenic mouse lines, we assessed the P301S-tau mice for mortality, morbidity, behavioral changes, and weight changes that could signal potential toxicity at the doses used here—0.3 and 1.0 mg/kg per day.

Most of the mice included in the study appeared and behaved normally. One P301S-tau female mouse receiving 1.0 mg/kg per day CLR01 showed limited movement, appeared dehydrated, and died shortly after the beginning of the treatment from what appeared to be a particularly aggressive course of the disease, not related to the treatment. Two male wild-type mice receiving 1.0 mg/kg per day CLR01 also died prematurely for unknown causes, one before completing the treatment and the other after completing the treatment, just before it was to be euthanized. As these were wild-type mice, their deaths were not related to an interaction of the compound with the transgene. The transgenic mice receiving 1.0 mg/kg CLR01 did not show a change in behavior, morbidity, or mortality for the duration of the treatment.

On average, the weight of the P301S-tau mice was ~ 10% lower than that of their wild-type littermates. The differences were more pronounced in the males compared to the females at all time points (Supplementary Figure S1) but there were no significant differences among the treatment groups, supporting the safety of the compound.

### CLR01 treatment protects P301S-tau mice from deterioration of muscle strength

**Fig. 2:**
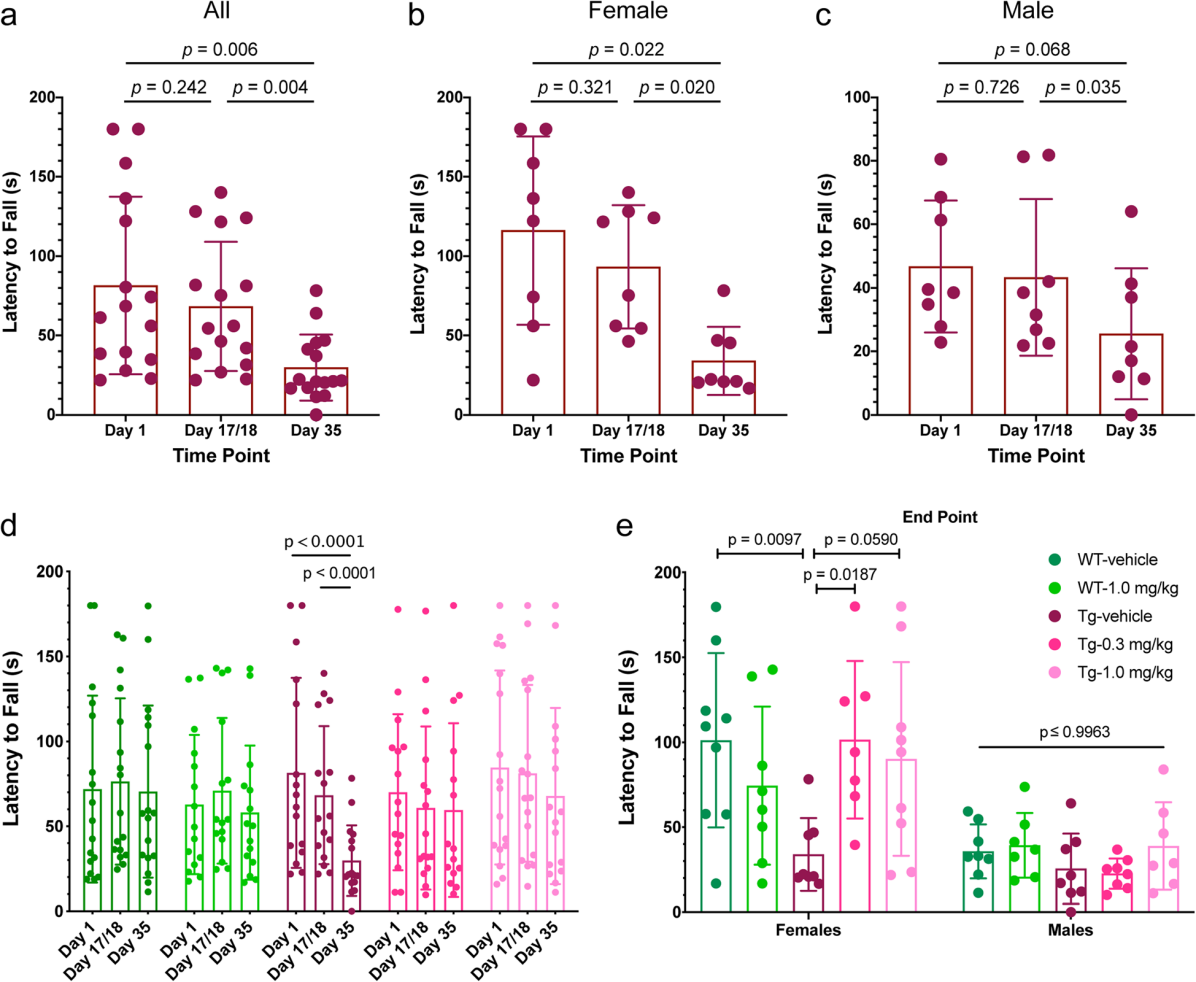
CLR01 protects P301S-tau mice from deterioration of muscle strength. P301S-tau and wild-type littermate mice were tested using the grip-strength assay on days 1, 17/18, and 35 of the treatment. **a** Treatment effect in all vehicle-treated P301S-tau mice. **b** Treatment effect in female, vehicle-treated, P301S-tau mice. **c** Treatment effect in male, vehicle-treated, P301S-tau mice. **d** Comparison of all treatment groups. **e** Comparison of the treatment groups, separated by sex, at the end of the experiment. The color key in panel E is for both panels **d** and **e**. The data in these two panels are presented as mean ± SD. *P* values were calculated using a one-way ANOVA (panels **a**–**c**), two-way, repeated-measure ANOVA (panel **d**), and two-way ANOVA (panel **e**) with post hoc Tukey test in all cases

Muscle-strength deficits are early signs of disease in the PS19 mouse model starting at ~ 7 months of age [43]. To test whether CLR01 treatment protected the mice from such deficits, we measured the muscle strength of the mice on day1, day 17/18, and day 35 of the treatment period using the grip-strength test [30]. After 17/18 days of treatment when the mice were 6.5–7 months old, the P301S-tau mice receiving vehicle showed a small decrease in their latency to fall from 81 ± 56 to 68 ± 41 s (Fig. 2a, *p* = 0.242, repeated-measure one-way ANOVA with post hoc Tukey test). During the second half of the treatment period, the mice deteriorated substantially and their latency to fall at the end of the treatment was 30 ± 21 (*p* = 0.006 compared to the start of the treatment). The deterioration was stronger in female mice (Fig. 2b) than in the males (Fig. 2c), possibly because the males’ latency to fall was substantially shorter than that of the females already at the beginning of the treatment due to their heavier weight.

CLR01 treatment prevented the deterioration in muscle strength in both treatment groups (Fig. 2d, e). Although in both groups the latency to fall decreased gradually from the beginning to the end of the treatment period, the differences were much smaller than in the vehicle-treated mice and, the combination of their small magnitude and associated *p* values suggested that these differences were not statistically meaningful (Supplementary Figure S2A, D). The differences between male and female mice were similar to those in the vehicle-treated group yet in both sexes and both treatment groups, the mice appeared to be protected from the decline in muscle strength (Supplementary Figure S2B, C, E, F). There were also no meaningful changes during the treatment in the muscle strength of wild-type mice receiving 1.0 mg/kg CLR01 per day compared to the group receiving vehicle (Fig. 2d and Supplementary Figure S3), further demonstrating that the treatment did not adversely affect the mice.

### CLR01 protects P301S-tau mice from disinhibition-like behavior

Disinhibition is a prominent behavioral feature of the behavioral variant of FTD (bvFTD) and often presents also in patients with AD as increased aggression, hyperactivity, and socially intrusive behavior [44–46]. An early-onset, progressive disinhibition-like behavior has been reported in the P301S-tau mice using the elevated-plus maze [47] together with early NFT pathology in the CA3 region, resembling clinical presentations and neuropathology of bvFTD.

The open-field test is a widely used method for assessing rodent behavior. Rodents show aversion to large, brightly lit, open, and unknown environments and perceive these types of environments as potentially dangerous. To determine whether the P301S-tau mice displayed genotype-related deficits, particularly disinhibition-like behavior, and whether CLR01 treatment could protect them from such deficits, we tested the mice in the open field-arena before the beginning of the treatment and on the last day of the treatment and recorded their exploration pattern and locomotion activity continuously over 5 min.

As the mice used in our study were relatively young and the open-field test is not physically or mentally challenging, some analyses did not show differences between the P301S-tau and wild-type mice, either before or at the end of the treatment, including overt anxiety-like behavior manifested as the time in the center or the ratio of time spent in the center relative to the periphery. Several measurements, including average speed, latency to enter the center zone, number of entries into the center, time spent in the center, number of line crossings, and path efficiency, increased or decreased substantially between the two measurements, but these changes were consistent across all the groups, regardless of genotype or treatment (data not shown).

The time spent mobile (before treatment—205 ± 36 s, after treatment—156 ± 49 s, Supplementary Figure S4A) and total distance traveled (before treatment—12.1 ± 0.6 m, after treatment—8.4 ± 1.5 m, Supplementary Figure S4B) decreased significantly between the two time points, likely reflecting the aging of the mice. This decrease was smaller in the P301S-tau mice than in the wild-type mice, so at the end of the treatment, the transgenic mice were ~ 35% more active in both measurements (Supplementary Figure S4, *p* = 0.001 for time mobile, *p* = 0.002 for distance traveled), suggesting that they were hyperactive [43]. CLR01 treatment had little effect on this phenotype (Supplementary Figure S4).

**Fig. 3:**
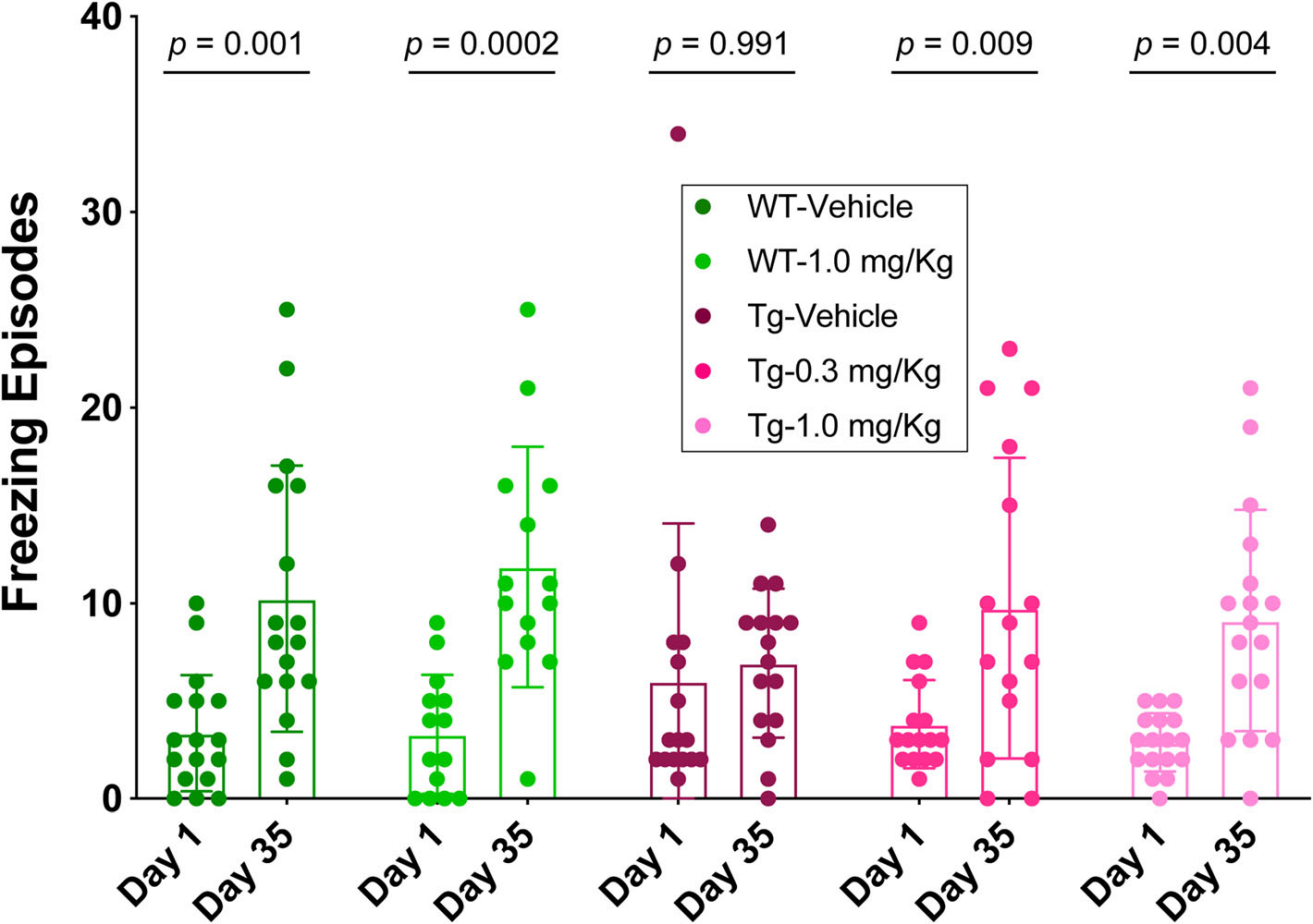
CLR01 protects P301S-tau mice from a disinhibition-like behavior. P301S-tau and wild-type littermate mice were examined using the open-field test for 5 min on days 1 and 35 of the treatment and the number of freezing episodes was recorded manually by a blinded operator. The data are presented as mean ± SD. *P* values were calculated using a mixed-effect, repeated-measure model with post hoc Sidak test

Freezing episodes during the open-field test may represent difficulty with movement and/or anxiety experienced by the mice. Because the mobility of P301S-tau mice at the end of the treatment increased relative to their wild-type littermates, we ruled out increased freezing due to movement difficulties and interpreted such observations as indicating anxiety. Comparing the number of freezing episodes at the beginning and end of the treatment, the wild-type mice treated with vehicle or 1.0 mg/kg per day exhibited a substantial increase from 3.4 ± 3.0 to 10.2 ± 6.8, *p* = 0.001, and from 3.3 ± 3.1 to 11.9 ± 6.2, *p* = 0.0002, respectively. In contrast, the P301S-tau mice receiving vehicle showed little change in freezing episodes, from 6.0 ± 8.1 to 6.9 ± 3.8, *p* = 0.991 (Fig. 3). Although the differences in the total number of freezing episodes among the groups were small, this lack of change was interpreted as a disinhibition-like behavior akin to the symptoms of bvFTD [48] and those reported previously in this mouse model [47]. Notably, one mouse in this group was an outlier, freezing 34 times during the test before the beginning of the treatment. When this outlier point was removed, the average number of freezing episodes in this group was 4.1 ± 3.2 and the *p*-value for the difference between the measurements before and after the treatment decreased to 0.419. The change in freezing episodes without the outlier was still substantially smaller in the vehicle-treated transgenic mice than in the wild-type mice. CLR01 treatment protected the mice from the disinhibition-like phenotype. The mice treated with 0.3 or 1.0 mg/kg per day showed increases in freezing episodes between the beginning and end of the treatment from 3.8 ± 2.3 to 9.8 ± 7.7, *p* = 0.009 and from 2.9 ± 1.5 to 9.1 ± 5.7, *p* = 0.004, respectively (Fig. 3).

The wild-type mice also showed a mild increase in grooming episodes between testing in the beginning and end of the treatment, a behavior suggesting discomfort. This change was not observed in P301S-tau mice treated with vehicle, but the P301S-tau mice treated with CLR01 showed a dose-dependent trend toward the difference seen in the wild-type mice (Supplementary Figure S5).

### Assessment of total tau in CNS-derived exosomes as a biomarker for treatment effect

**Fig. 4:**
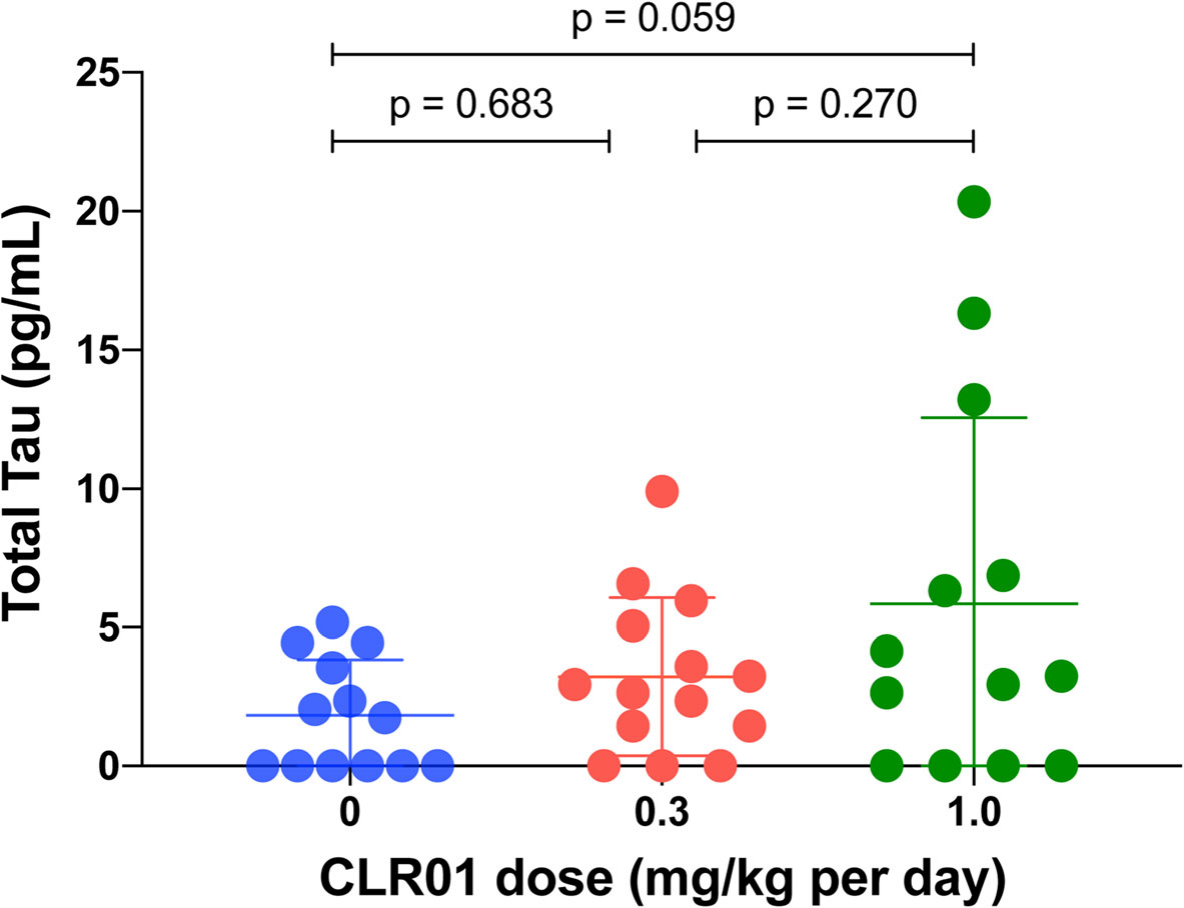
CLR01 treatment increases tau release in neuronal exosomes isolated from the blood. Blood was collected from P301S-tau mice at the point of sacrifice, serum was separated, and neuronal exosomes were isolated by immunoprecipitation using an anti-mouse L1CAM antibody. Exosomes were lysed in RIPA buffer and the concentration of tau was measured by an ELISA specific for human tau. The data are presented as mean ± SD. P-values were calculated using a one-way ANOVA with post hoc Tukey test

Analysis of biomarkers in CNS-derived exosomes isolated from the blood is a new approach that offers a minimally invasive, unique window into biochemical changes in the brain [49, 50]. This methodology has been applied to the measurement of tau in neuronal exosomes from patients with AD, FTD, and other diseases [51–54]. However, how drugs targeting tau aggregation, such as CLR01, affect the concentration of tau in such exosomes is not known. To address this question, we collected the blood of the mice at sacrifice, separated the serum, and enriched neuronal exosomes from the serum by immunoprecipitation using magnetic beads conjugated to a monoclonal antibody against the neuronal marker L1CAM [50]. After lysis of the exosomes, the concentration of total tau was measured using ELISA. The small volume of blood that could be collected from each mouse (100–150 μL) did not allow measurement of additional biomarkers. The measurement showed a dose-dependent increase from 1.8 ± 2.0 pg/mL in the vehicle-treated mice to 3.2 ± 2.9 and 5.9 ± 6.7 pg/mL in the low- and high-dose CLR01 groups, respectively (Fig. 4). The high variability observed precluded making definite conclusions regarding the utility of this assay for assessment of treatment effect, yet the data suggested that measurement of tau in CNS-derived exosomes could be useful in human studies, where larger volumes of blood are easily attainable, and possibly using higher-sensitivity methods for biomarker measurement than the ELISA we used here.

### CLR01 reduces hyperphosphorylated tau in the hippocampus

**Fig. 5:**
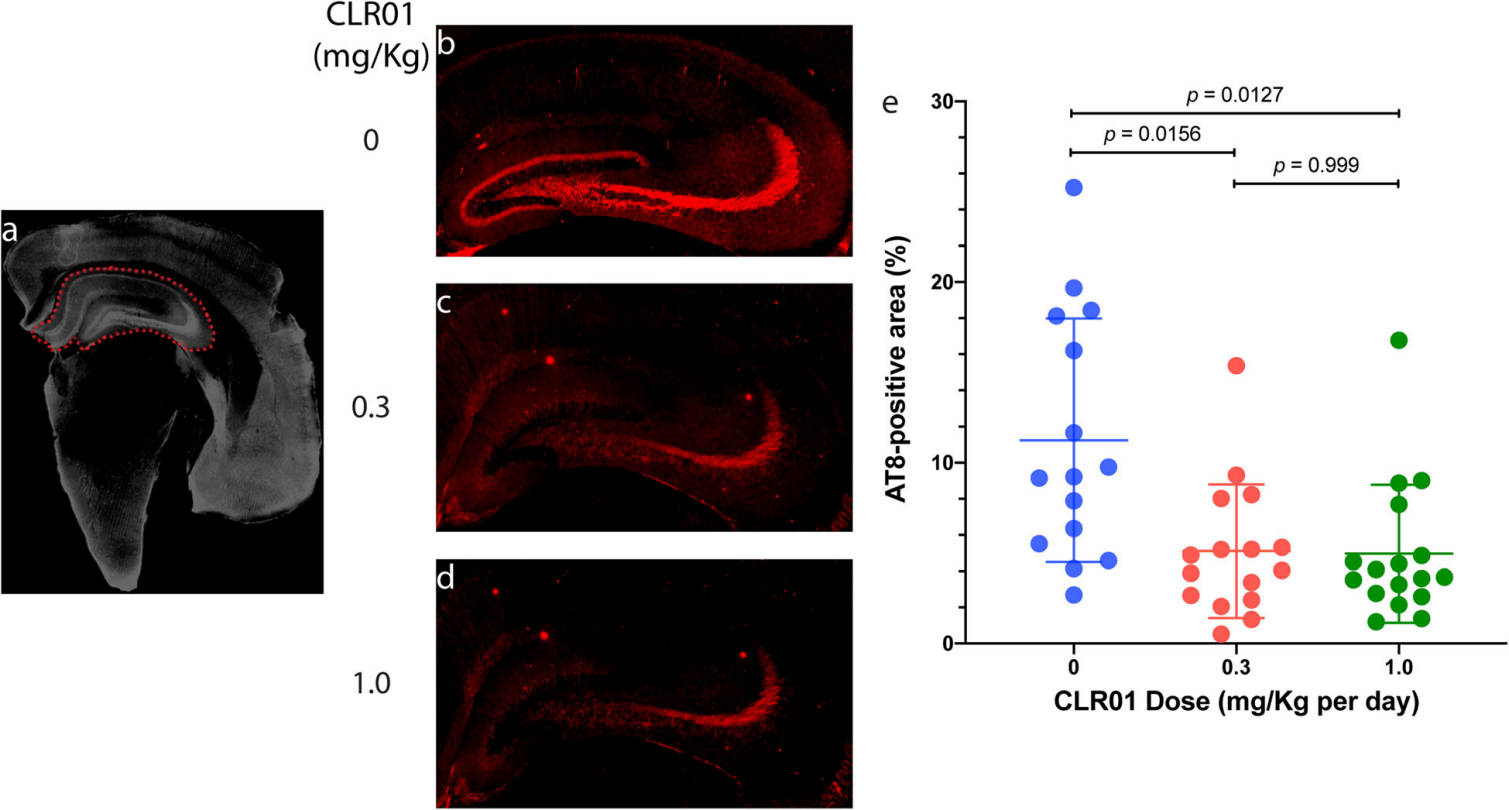
CLR01 treatment reduces hyperphosphorylated tau in the hippocampus of P301S-tau mice. Brain sections from P301S-tau mice were stained with monoclonal antibody AT8 and visualized by immunofluorescence. **a** The typical crescent shape of the hippocampus was delineated by an operator blinded to treatment. **b**–**d** Representative images of the hippocampus area of mice treated with 0 (**a**), 0.3 (**b**), or 1.0 (**c**) mg/kg per day CLR01. **d** The data were quantified as the percentage of AT8-positive area within the hippocampus area, as defined in panel **a**. The data are presented as mean ± SD. *P* values were calculated using a one-way ANOVA with post hoc Tukey test

After completing the behavioral tests and sacrificing the animals, we analyzed their brain pathology using several histological and immunohistochemical stains. Tau hyperphosphorylation in the hippocampus was analyzed using monoclonal antibody AT8, which detects specifically phosphorylation at Ser202 and Thr205, two major sites of tau phosphorylation in paired helical filaments in the AD brain [55, 56]. The typical crescent shape of the hippocampus was delineated by operators blinded to treatment (Fig. 5a). The vehicle-treated transgenic mice showed abundant AT8 staining in the hippocampus, primarily in the CA3 region, covering 11.2 ± 6.7% of the area (Fig. 5b,e). CLR01 treatment reduced the staining to 5.1 ± 3.7% (*p* = 0.0156) and 5.0 ± 3.8% (*p* = 0.0127) in the 0.3 and 1.0 mg/kg per day groups (Fig. 5c–e), suggesting that a maximal effect was reached already at the low dose. Analysis by sex showed that the results were driven mainly by the male mice, which showed substantially higher AT8 reactivity in the hippocampus compared to the female mice (Supplementary Figure S6).

### CLR01 treatment reduces tau aggregates in the CA3 region

**Fig. 6:**
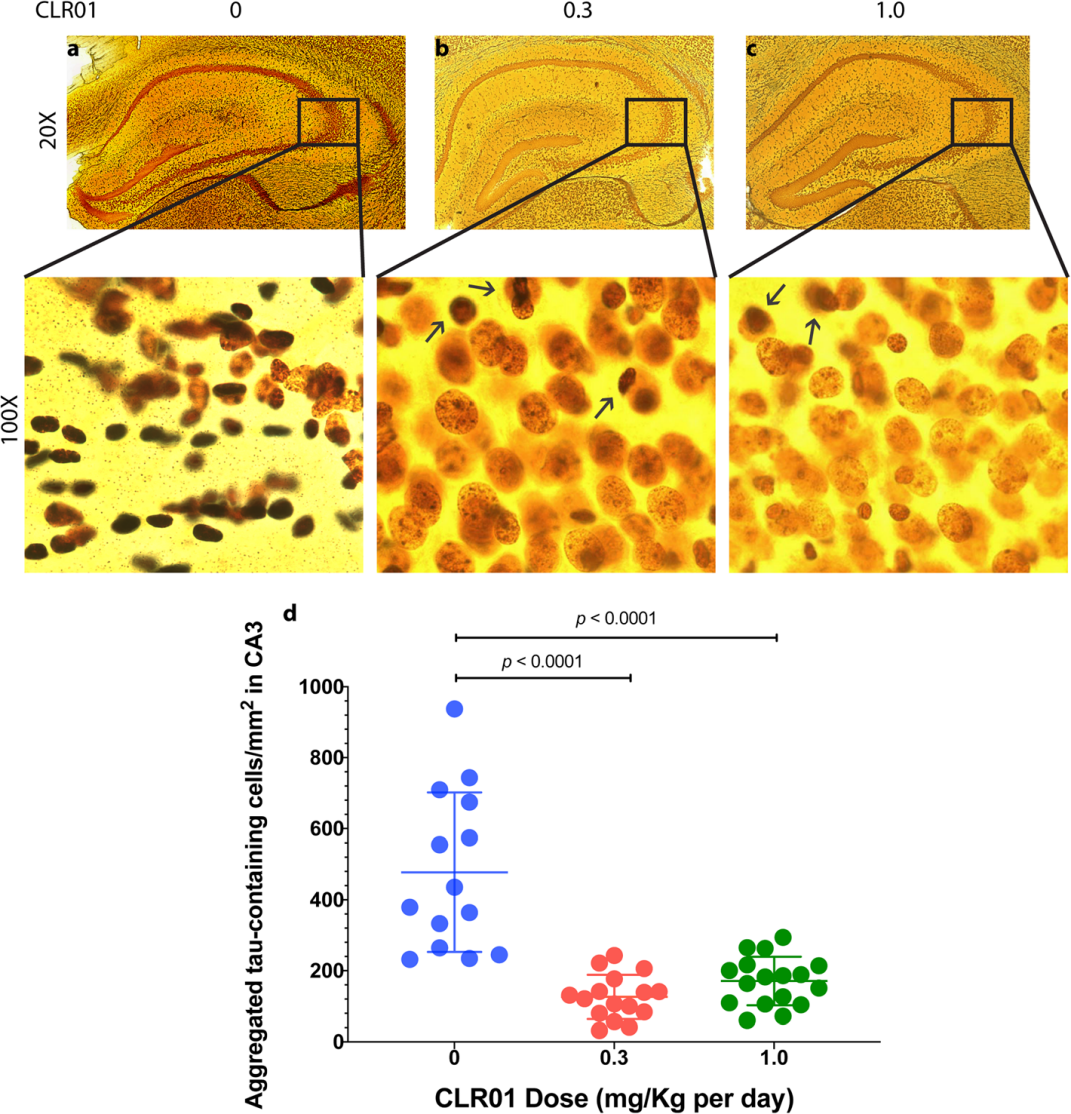
CLR01 treatment reduces tau aggregation in the CA3 region of P301S-tau mice. Brain sections from P301S-tau mice were stained with Gallyas silver-stain. **a**–**c** Representative images of the hippocampus (top) and zooming on the CA3 region (bottom) are presented for the mice treated with 0 (**a**), 0.3 (**b**), or 1.0 (**c**) mg/kg per day CLR01 and show black/dark brown cells containing aggregated tau. **d** The data were quantified as the number of tau-aggregate-containing cells per mm^2^ in the CA3 and are presented as mean ± SD. *P* values were calculated using a one-way ANOVA with post hoc Tukey test

In view of the predominant hyperphosphorylated tau pathology observed in the CA3 region, subsequent histological analyses focused on this region. Gallyas silver-staining is a classical histologic method for identification of pathological tau deposits [57]. Black or dark brown cells indicate a large amount of protein deposition, whereas yellow/light brown cells are healthy and devoid of abnormal protein deposition. Our analysis showed abundant black deposits in the CA3 region of vehicle-treated P301S-tau mice, whose morphology suggested that they were dead cells filled with tau deposits or “ghost” protein deposits left behind after the cells died (Fig. 6a). In contrast, in the CA3 region of mice treated with 0.3 (Fig. 6b) or 1.0 (Fig. 6c) mg/kg per day CLR01, most of the cells appeared to be healthy and tau deposition was observed only in a few cells (Fig. 6b, c, arrows). We quantified the data as the number of cells containing aggregated tau per mm^2^. The vehicle-treated group had 477 ± 244 of these cells per mm^2^ (Fig. 6d), whereas the low-dose group had 126 ± 62 cells per mm^2^ (*p* = 0.0001 compared to the vehicle-treated mice), and the high-dose group had 171 ± 68 aggregated-tau-containing cells per mm^2^ (*p* = 0.0005 compared to the vehicle-treated mice), demonstrating again that the full effect of the treatment on aggregated tau deposition was achieved already at 0.3 mg/kg per day CLR01. Analysis of male and female mice separately showed again that the main difference was in the males. This was because the vehicle-treated males had substantially a higher number of aggregated-tau-containing cells in the CA3 (651 ± 176) compared to the females (303 ± 80, *p* < 0.0001, Supplementary Figure S7).

In view of the silver-stain data presented above, we asked if overt neurodegeneration might be observed in the mice. Previously, neurodegeneration in the PS19 mouse model has been reported at 9 months of age [58] and the mice used in our study were sacrificed at age 7–7.5 months. Nonetheless, we reasoned that if early signs of neurodegeneration could be observed, they would allow assessing a potential protective effect of CLR01 treatment against neurodegeneration. For this assessment, brain sections were stained using a monoclonal antibody against the neuronal nuclear marker NeuN. The analysis showed neurodegeneration in the CA3 region of two mice in the vehicle-treated group, which was accompanied by apparent astrogliosis (Supplementary Figure S8A), yet other mice in this group did not show neurodegeneration, likely due to the relatively young age of the mice. Neurodegeneration was not observed in either of the treatment groups (Supplementary Figure S8b, C), yet the lack of an overt phenotype in the vehicle-treated group precluded meaningful analysis of treatment effect (Supplementary Figure S8D.)

We also analyzed synapse density in the CA3 region using monoclonal antibodies against the presynaptic marker Bassoon and postsynaptic marker Homer. The analysis showed that the P301S-tau mice had ~ 50% reduction in synapse density in the CA3, which was only marginally affected by the treatment (Supplementary Figure S9). Impaired synaptic function and hippocampal synapse loss have been detected in the P301S mouse as early as 3 months of age [28], 3–3.5 months before the beginning of the treatment, at which point substantial synapse loss was already present and apparently could not be reversed by the treatment.

### CLR01 treatment effect on hyperphosphorylated tau in different brain regions of P301S-tau mice

To test whether the CLR01 treatment affected the levels of total and hyperphosphorylated tau in different brain regions of the treated P301S-tau mice, we compared the fraction of total tau that was phosphorylated at pT231, each measured by ELISA, among the treatment groups. Total tau was normalized to the total protein in each sample. Tau contains 85 potential phosphorylation sites but not all of them become phosphorylated in disease and contribute to the pathology. We measured pT231-tau as phosphorylation at this site is associated with most tauopathies and decreases the ability of tau to stabilize microtubules [59].

**Fig. 7:**
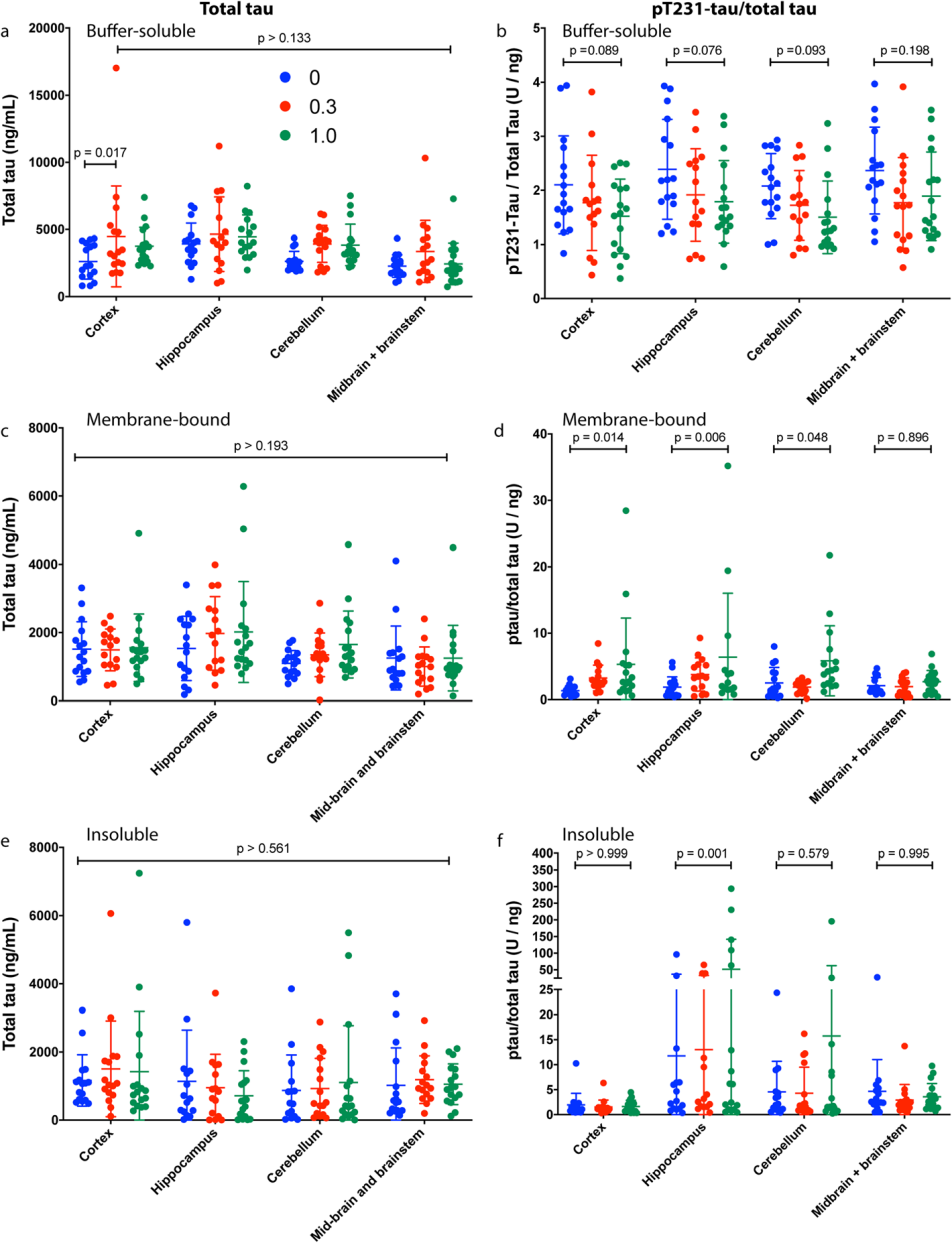
CLR01 treatment alters the pT231-tau/total tau ratio in the brain of P301S-tau mice. Total-tau (**a**, **c**, **e**) and pT231-tau/total tau (**b**, **d**, **f**) were measured in the soluble (**a**, **b**), membrane-bound (**c**, **d**), and insoluble (**e**, **f**) fractions of four brain-region extracts. The data are presented as mean ± SD. *P* values were calculated using a two-way ANOVA with post hoc Tukey test

Measurement of total tau in the buffer-soluble fraction showed that the concentration was slightly higher in the CLR01-treated groups compared to the vehicle group, yet the differences were small and in almost all cases statistically insignificant (Fig. 7a). Similar small, insignificant changes were observed in the membrane-bound (Fig. 7c) and insoluble (Fig. 7e) fractions, in which the concentration of tau was substantially lower than in the buffer-soluble fractions.

The fraction of pT231-tau in total tau in the soluble fraction decreased in all the brain regions analyzed in a dose-dependent manner, except in the midbrain/brainstem extract where the decrease in the low-dose group was more pronounced than in the high-dose group (Fig. 7b), reflecting high experimental variability in these measurements. Interestingly, in the membrane-bound fraction of the cortex, hippocampus, and cerebellum, but not the midbrain/brainstem, we found a dose-dependent increase in the fraction of pT231-tau in total tau (Fig. 7d). A similar increase was found in the insoluble fraction of the hippocampus, though it appeared to be driven by a few samples in which the pT231-tau fraction in total tau was 1–2-orders of magnitude higher than in most other samples (Fig. 7f) and is therefore difficult to interpret.

### CLR01 reduces tau oligomers in the hippocampus dose-dependently

**Fig. 8:**
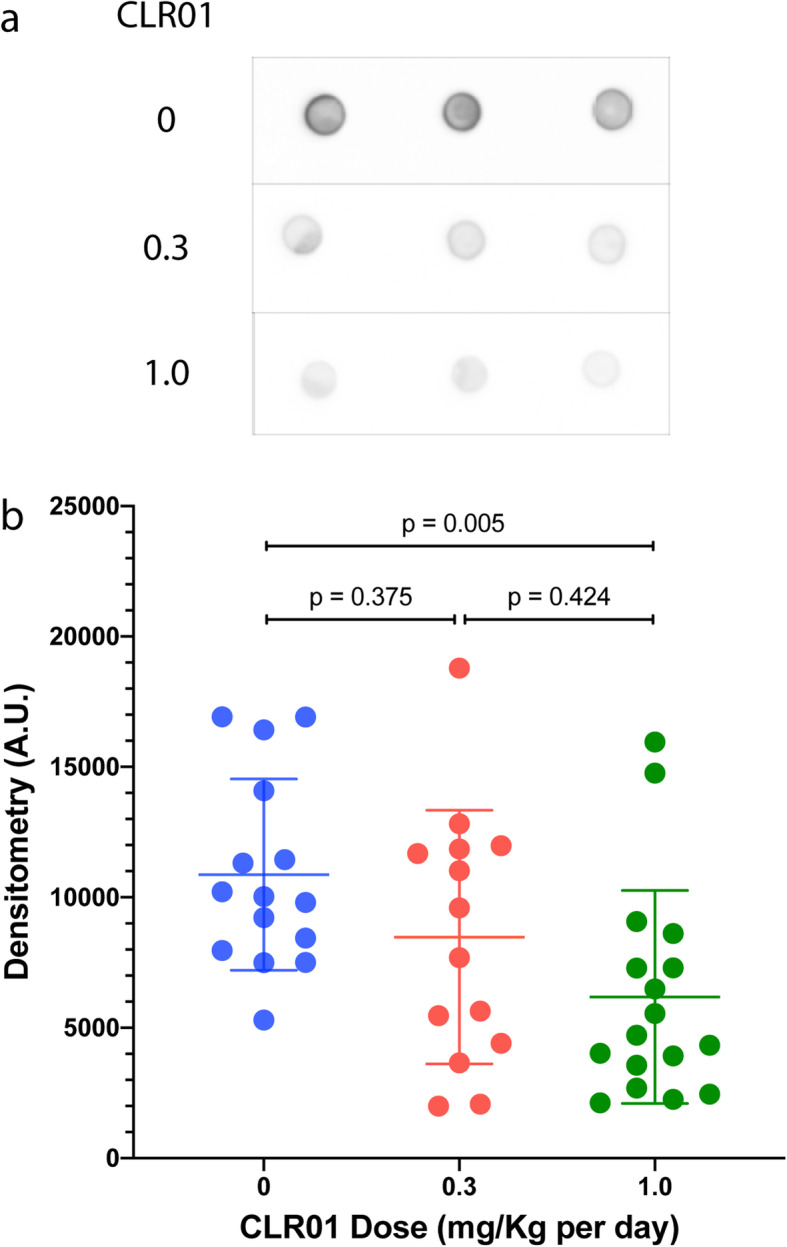
CLR01 treatment reduces tau oligomers in the hippocampus of P301S-tau mice. The soluble fraction from the hippocampus of P301S-tau mice was examined by dot blots probed using the anti-tau-oligomer antibody TOC-1. **a** Representative dot blots showing the analysis of one mouse from each group in triplicates. **b** The data were quantified by densitometry and are presented as mean ± SD. *P* values were calculated using a one-way ANOVA with post hoc Tukey test

Tau oligomers often are considered the most toxic form of the protein [60, 61]. Mouse studies have shown that memory loss and synapse loss correlated better with oligomer concentration levels than with the amount or spreading of NFTs [62, 63]. In view of these data, we tested whether CLR01 treatment affected tau-oligomer levels using dot-blots and native-PAGE/western-blot analyses in the soluble fraction of the brain extracts. The other two fractions were not used because the detergent or chaotrope in their buffers likely alter the oligomer composition. We probed the dot blots using monoclonal antibody tau oligomeric complex-1 (TOC-1) [64]. We did not detect any TOC-1 signal in the cortex, cerebellum, or midbrain/brainstem regions even when the total protein amount loaded was 9 μg, three times higher than in the hippocampus (data not shown). In contrast, a strong TOC-1 signal was detected in the hippocampus extracts when 3 μg of total protein were loaded, suggesting that TOC-1-positive tau oligomers were present primarily or only in the hippocampus. The analysis showed a dose-dependent decrease in the TOC-1 signal (Fig. 8a). Densitometric quantitation of the dot blots showed a relatively high degree of variability within each treatment group. The densitometric signal in the vehicle-treated mice was 10,871 ± 3664 and was reduced to 8475 ± 4863 (*p* = 0.375 compared to the vehicle-treated group) and 6182 ± 4082 (*p* = 0.005) in the 0.3 and 1.0 mg/kg per day CLR01 groups, respectively (Fig. 8b). Analysis of male and female mice separately showed similar trends in both sexes (Supplementary Figure S10A).

We also attempted to quantify individual tau oligomers by native-PAGE/western blots probed using the anti-human tau monoclonal antibody HT7. However, high variability among the mice yielded little differences among the groups and the results were not statistically meaningful (*p* value range 0.107–0.996). An example of the analysis in the hippocampus is shown in Supplementary Figure S10B. Analyses in other brain regions yielded similar results (not shown).

### CLR01 reduces the concentration of tau seeds in the hippocampal soluble fraction

Intercellular propagation of proteopathic protein seeds is thought to be a major mechanism by which tau pathology propagates in the brain in a prion-like manner [65–67]. Therefore, we asked if the reduction of aggregated, hyperphosphorylated, and oligomeric tau observed in the P301S-tau mice treated with CLR01 correlated with a reduction in the concentration of tau seeds. We addressed this question by measuring seeding activity in the soluble and insoluble fractions using a HEK293 biosensor cell line that expresses stably the 4R-tau repeat domain containing a P301S substitution and conjugated to CFP or YFP [68]. The detergent present in the membrane-bound fraction is toxic to the cells, preventing the use of this fraction in the assay. In each experiment, the cells were imaged by fluorescence microscopy using the fluorescence of YFP to obtain a qualitative assessment of the seeding response, and the number of seeds was quantified by measuring the FRET signal between CFP and YFP using flow cytometry, as described previously [26].

**Fig. 9:**
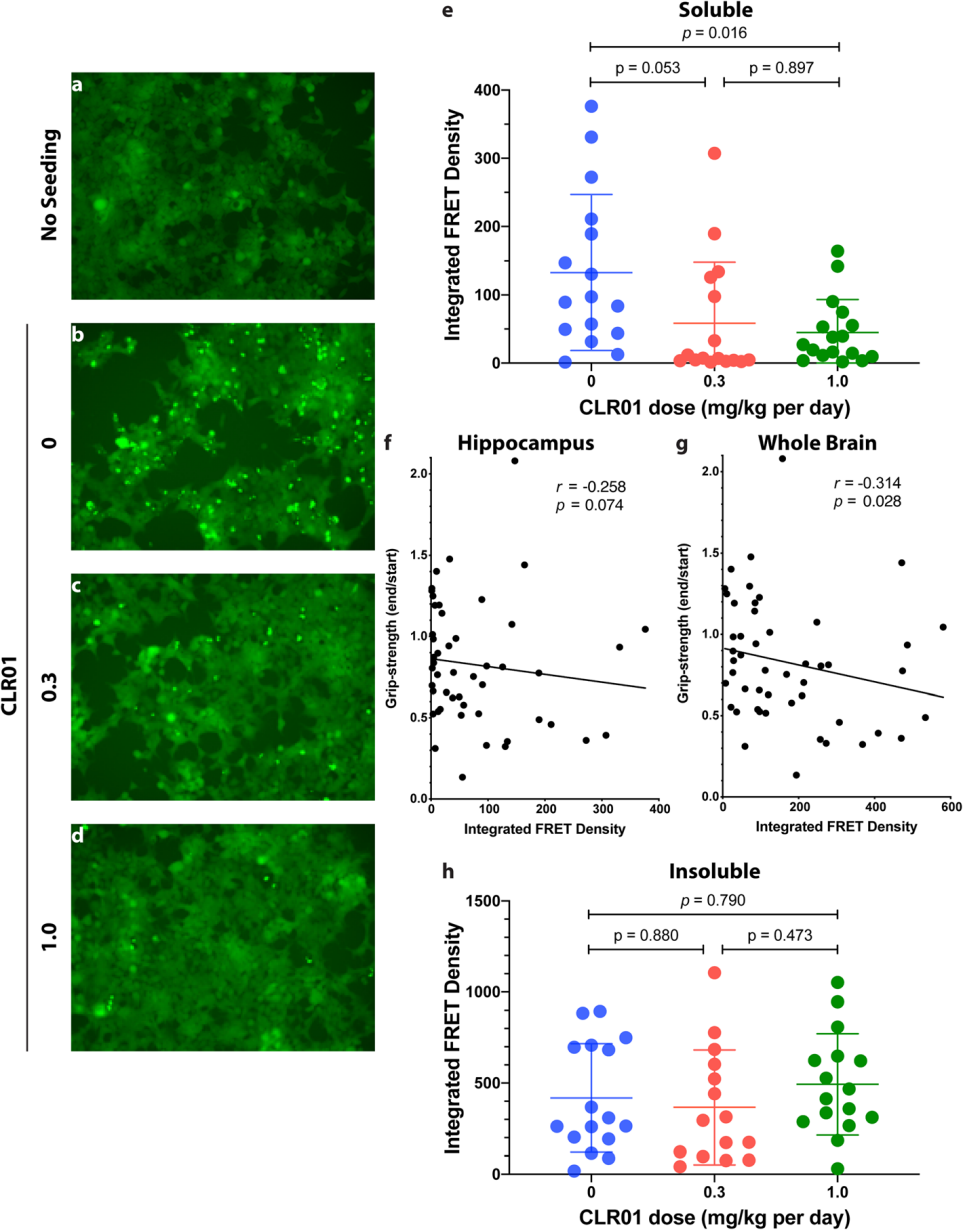
CLR01 treatment reduces tau seeding of hippocampal brain extracts in tau-biosensor cells. The soluble or insoluble fraction of brain extracts from P301S-tau mice were added to tau-biosensor cells and incubated for 48 h. The cells were visualized by a fluorescence microscope and their integrated FRET density was measured by flow cytometry. **a**–**d** Representative micrographs of **a** control, unseeded biosensor cells. **b** Biosensor cells seeded with the soluble fraction of a mouse in the vehicle-treated group. **c** Biosensor cells seeded with the soluble fraction of a mouse in the low-dose group. **d** Biosensor cells seeded with the soluble fraction of a mouse in the high-dose group. **e** Integrated FRET density analysis of the soluble fraction. **f** Spearman correlation between grip-strength and integrated FRET density in the hippocampus. **g** Spearman correlation between grip-strength and integrated FRET density in the whole brain (calculated by adding the values measured for the different brain regions). **h** Integrated FRET density analysis of the insoluble fraction. The data in panels **e** and **h** are presented as mean ± SD. *P* values were calculated using a one-way ANOVA with post hoc Tukey test

Examples of the fluorescence-microscopy images are shown for the soluble fraction of the hippocampus. In the absence of seeds, only diffuse fluorescence was observed (Fig. 9a), whereas in the presence of an extract from a vehicle-treated mouse, abundant bright puncta were apparent, indicating the formation of intracellular tau aggregates (Fig. 9b). The aggregates were substantially decreased in the presence of extracts from mice treated with 0.3 or 1.0 mg/kg per day CLR01 (Fig. 8c, d, respectively).

The FRET-based flow-cytometry analysis showed a marked decrease in the seeding activity (expressed as integrated FRET density) of hippocampal extracts from 133 ± 114 in the vehicle-treated P301S-tau mice to 59 ± 90 (*p* = 0.053) and 45 ± 48 (*p* = 0.016) in the low- and high-dose groups, respectively (Fig. 9e). Both the male and the female mice contributed to these differences (Supplementary Figure S11A). To further explore whether the presence of tau seeds in the brain of the mice had a deleterious effect, we asked whether the seeding correlated with the ratio between the grip-strength at the end versus the beginning of the treatment. Spearman correlation showed an increase in the seeding response of the hippocampal extract correlated with a stronger decline in grip-strength (Fig. 9f; *r* = − 0.258, *p* = 0.074). Stronger correlations were found in extracts of the cerebellum (Supplementary Figure 11F) and midbrain/brainstem (Supplementary Figure 11I), which are important brain regions for control of movement, but not in the cortex (Supplementary Figure 11C). The correlation was strong also for all the brain regions combined (Fig. 9g; *r* = − 0.314, *p* = 0.028). In contrast to the impact of the treatment on the seeds in the soluble fraction, the treatment had no apparent effect on the amount of seeds in the insoluble fraction (Fig. 9h).

Despite the contribution of other brain regions to the increased correlation between the seeds and the grip-strength deterioration (Fig. 9g), the seeding response of the soluble fraction of the cortex (Supplementary Figure S11B), cerebellum (Supplementary Figure S11E), and midbrain/brainstem (Supplementary Figure S11H), which was substantially lower than that of the hippocampus, was largely unaffected by the treatment. The seeding responses of the insoluble fractions of the same regions were higher than those of the soluble fractions but were not affected by the treatment (Supplementary Figure S11D, G, J).

### CLR01 reduces microgliosis in P301S-tau mice

**Fig. 10:**
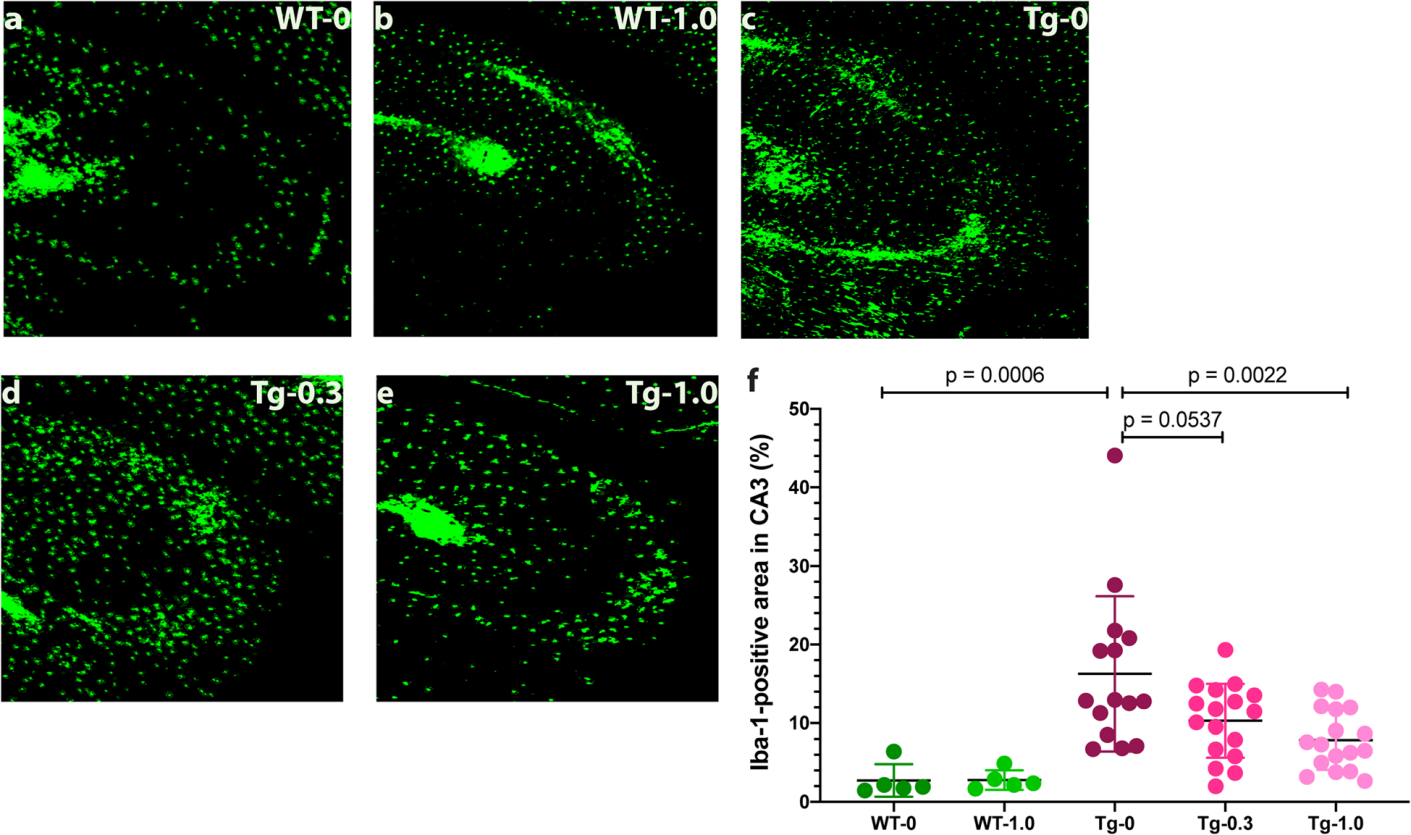
CLR01 treatment reduces microglia density in the CA3 region of P301S-tau mice. Brain sections from P301S-tau mice and wild-type littermates were stained using an anti-Iba-1 antibody and visualized by immunofluorescence. **a**–**e** Representative images of the CA3 area of wild-type (WT) mice treated with 0 (**a**) or 1.0 (**b**) mg/kg CLR01 and transgenic (Tg) P301S-tau mice treated with 0 (**c**), 0.3 (**d**), or 1.0 (**e**) mg/kg per day CLR01. **f** The data were quantified as the percentage of Iba-1-positive area in the CA3 and are presented as mean ± SD. *P* values were calculated using a one-way ANOVA with post hoc Tukey test

Glial cells, including microglia and astrocytes, are major contributors to the neuropathological processes, neuroinflammation, and spreading of pathology in tauopathies [69, 70]. To assess the impact of CLR01 treatment on glial involvement in the developing neuropathology in the P301S-tau mouse brain, we stained the brains using antibodies against the microglial and astrocytic markers ionized calcium-binding adaptor molecule 1 (Iba-1) and glial fibrillary acidic protein (GFAP), respectively. Compared to wild-type mice treated with vehicle (2.7 ± 2.1% of the CA3 area, Fig. 10a, f) or 1.0 mg/kg per day CLR01 (2.8 ± 1.3%, Fig. 10b, f), microglia were found to be densely populated in CA3 region of the vehicle-treated P301S-tau mice (16.3 ± 9.9% Fig. 10c, f; *p* = 0.0006 compared to vehicle-treated wild-type mice). The density was substantially reduced in the low- (10.3 ± 4.7%, *p* = 0.054 compared to vehicle-treated P301S-tau mice, Fig. 10c, f) and high-dose (7.9 ± 3.8%, *p* = 0.002, Fig. 10d, f) CLR01 treatment groups. The treatment effect was driven primarily by the male mice, whereas in the females, a similar trend was observed but the differences among the groups were much smaller (Supplementary Figure S12A).

A peculiar phenomenon we observed in the cortex of two female mice in the vehicle-treated P301S-tau group while analyzing the microglia was clustering of the cells at apparently random spots (Supplementary Figure S12B). The cells appeared much larger than neighboring microglia and their focal point did not appear to be hyperphosphorylated tau.

In contrast to the substantial differences in microglia density, the differences in astrocyte density between the wild-type and transgenic mice and the impact of CLR01 on the astrocyte density showed a similar trend but were of smaller magnitude and statistically insignificant (Supplementary Figure S13).

## Discussion

There is no disease-modifying therapy for tauopathies to date. Several strategies have been tested in animal models and a few of them reached clinical trials. Among the most common strategies are modulating post-translational modifications of tau, including phosphorylation, O-linked glycosylation, and acetylation, inhibiting proteolytic processing of tau, improving cellular proteostasis, modulating tau-expression, decreasing microtubule dynamics, active and passive immunotherapy, and inhibition of tau aggregation [71, 72]. Here, we tested our lead molecular tweezer, CLR01, a broad-spectrum, small-molecule inhibitor of protein aggregation that disrupts a specific combination of electrostatic and hydrophobic interactions, in a pure tauopathy model. The goal of our study was to determine whether CLR01 could inhibit tau aggregation, toxicity, and spreading by a direct interaction with the protein, rather than as a secondary effect through inhibition of Aβ self-assembly. Our results demonstrate that the answer is a resounding yes. CLR01 treatment in the P301S-tau model ameliorated muscle strength deterioration (Fig. 2 and Supplementary Figure 3) and disinhibition-like behavior (Fig. 3), and reduced tau hyperphosphorylation (Fig. 5 and Supplementary Figure S6), aggregation (Fig. 6 and Supplementary Figure S7), oligomerization (Fig. 8 and Supplementary Figure S10A), and seeding capacity (Fig. 9 and Supplementary Figure S11A) in the brain of the P301S-tau mice. The treatment also reduced microglial density in the CA3 region of the hippocampus, suggesting reduced brain inflammation (Fig. 10 and Supplementary Figure S12A).

Due to high experimental variability among the mice, several treatment effects were found as trends that did not reach high level of statistical significance, but nonetheless supported the overall therapeutic effect of CLR01 in these mice, including a reduced change in grooming (Supplementary Figure S5), the fraction of pT231-tau in total tau (Fig. 7b), and astrocyte density in the CA3 region (Supplementary Figure 13).

Analysis of the effect of sex showed that in most cases male mice had a more severe phenotype and therefore the treatment effect was larger, in most cases, in the males (Supplementary Figures S6, S7, S11A, and S12A), in agreement with a more severe phenotype reported in male mice in this model [73]. An exception was the grip-strength test, in which female P301S-tau mice showed a stronger deterioration in the second half of the treatment period and a stronger rescue effect, likely because the heavier male mice had difficulty holding on to the wire mesh and fell in most cases within 40–60 s.

In general, we found stronger effects of CLR01 on pathological forms of tau, such as pT231-tau (Fig. 7b), TOC-1-positive oligomers (Fig. 8), and tau seeds (Fig. 9a–g) in the soluble fraction than in the membrane-bound or insoluble fractions (Figs. 7d, f and 9h). This difference is expected based on the mechanism of action of CLR01, which involves highly labile binding to Lys (and to a lower extent to Arg) residues. The binding has a high on-off rate because the activation barrier of the association and dissociation of the complex between Lys and CLR01 is low so that both processes are fast at room temperature. This mode of binding interferes efficiently and selectively with weak forces, such as those mediating the formation of oligomers and seeds, without affecting normal, physiologic, protein–protein interactions [15, 42]. However, although the gentle binding of CLR01 is not expected to have a strong effect on pre-formed fibrillar aggregates directly, as has been shown in vitro for several amyloidogenic proteins [18, 74, 75], in vivo, the compound facilitates clearance of the offending proteins by allowing clearance mechanisms that are shut down by the toxic oligomers and aggregates to resume their function. This has been demonstrated for the proteasome in a zebrafish embryo model of α-synucleinopathy [74] and more recently in a mouse model of the lysosomal-storage disease, mucopolysaccharidosis type IIIA [40]. 

Biomarkers for tauopathies currently are an urgent, unmet public-health need. PET ligands specific for NFTs in the AD brain recently have been developed, yet limitations including high costs, need for specialized instrumentation, and lack of binding to different tau strains [76, 77] suggests that fluid biomarkers, especially those obtained by minimally invasive procedures, such as a blood test, may be complementary or even advantageous to imaging biomarkers. A recent example is plasma pT217-tau, which recently has been shown to distinguish AD from other neurodegenerative diseases with high sensitivity and specificity [78, 79]. Here, we used neuronal exosomes enriched from the serum to test total tau concentration as an outcome measure for the treatment. Though the variability was high and the low blood volume collected did not allow measuring potentially more informative biomarkers, such as different phosphorylated forms of tau or neurofilament light chain, the observed dose-dependent increase in tau concentration in the exosomes (Fig. 4) suggests that the strategy of measuring biomarkers in CNS exosomes isolated from blood is a promising surrogate outcome measure for treatment effect. The data also suggest that one mechanism by which the treatment might have enhanced tau clearance was via packaging and release of the protein in exosomes. In addition, as tau is not typically considered a membrane-associated protein, the increase in exosomal tau with CLR01 dose also may explain the increase in membrane-associated tau and pT231-tau observed in several brain regions (Fig. 7c, d), though at this point this is only conjecture.

## Limitations

The P301S-tau mouse model shows a high variability in disease progression and severity, which in several cases resulted in a high *p* value and limited our ability to obtain conclusive data for some of the outcome measures, as discussed above. An additional limitation of the study was the relatively short duration and the early age of the mice. The age and treatment duration were conservative choices designed to limit costs while providing an unequivocal answer to the question whether CLR01 can ameliorate tau pathology directly in vivo, in the absence of Aβ. Finally, the delivery of the drug via osmotic pumps, which was chosen for the same reasons, limits translatability to humans. As discussed above, recent studies have demonstrated that continuous administration is unnecessary and subcutaneous administration of CLR01 daily [38, 40], or even twice a week can result in a robust therapeutic effect [41].

## Conclusion

Our findings suggest that CLR01 is a promising drug candidate for the prevention and possibly treatment of AD and other tauopathies. The ability of CLR01 to target directly both Aβ and tau, and the fact that it can also inhibit formation of toxic assemblies by other amyloidogenic proteins involved in these diseases, such as α-synuclein [32, 36, 41] and TDP-43 (unpublished data), make it a particularly attractive compound. Our results demonstrate that peripherally administered CLR01 reduces tau hyperphosphorylation, oligomerization, and aggregate formation in the brain, as well as the formation of tau seeds. As a result, CLR01 reduces early behavioral deficits in the P301S-tau model, suggesting that similar therapeutic effects could be translated to human therapy.

## Supplementary Information

**Additional file 1.**

## Data Availability

The raw data used in preparation of this manuscript are available upon request.

## Abbreviations

AD: Alzheimer’s disease
Aβ: Amyloid β-protein
AGD: Argyrophilic grain disease
APP: Amyloid β-protein precursor
BB: Blocking buffer
BCA: Bicinchoninic acid
BSA: Bovine serum albumin
CBS: Corticobasal syndrome
bvFTD: Behavioral variant of frontotemporal dementia
GFAP: Glial fibrillary acidic protein
Iba-1: Ionized calcium-binding adaptor molecule-1
MAPT: Microtubule-associated protein tau
MT: Molecular tweezer
NFT: Neurofibrillary tangles
PBS: Phosphate-buffered saline
PD: Parkinson’s disease
PPI: Protease and phosphatase inhibitor cocktail
Prnp: Prion protein
PS1: Presenilin 1
PSP: Progressive supranuclear palsy
RIPA: Radioimmunoprecipitation assay
s.c.: Subcutaneous
TBS: Tris-buffered saline
3 × Tg: Triple transgenic
TOC-1: Tau oligomeric complex 1
