# Correction to: The molecular tweezer CLR01 improves behavioral deficits and reduces tau pathology in P301S-tau transgenic mice

**DOI:** 10.1186/s13195-021-00823-6

**Published:** 2021-04-22

**Authors:** Jing Di, Ibrar Siddique, Zizheng Li, Ghattas Malki, Simon Hornung, Suman Dutta, Ian Hurst, Ella Ishaaya, Austin Wang, Sally Tu, Ani Boghos, Ida Ericsson, Frank-Gerrit Klärner, Thomas Schrader, Gal Bitan

**Affiliations:** 1 Department of Neurology, David Geffen School of Medicine, University of California, Gordon Neuroscience Research Building, Room 451, 635 Charles E. Young Drive South, Los Angeles, CA 90095-7334 USA; 2 Present address: Division of Peptide Biochemistry, Technical University of Munich, Freising, Germany; 3 Faculty of Chemistry, University of Duisburg-Essen, Essen, Germany; 4 Brain Research Institute, University of California, Los Angeles, CA USA; 5 Molecular Biology Institute, University of California, Los Angeles, CA USA

**Correction to: Alzheimers Res Ther 13, 6 (2021)**

**https://doi.org/10.1186/s13195-020-00743-x**

Following publication of the original article [1], the authors reported an error in Figure 5 and Supplementary Figure 9. In Figure 5, the same image was included, by mistake, in panels c and d. The corrected Figure 5 shows the correct image in panel c. Similarly, in Supplementary Figure 9, panels G and K show, by mistake, the same image. The corrected Supplementary Figure 9 shows the correct image in panel G, presented below as Fig. 1.

**Fig. 1:**
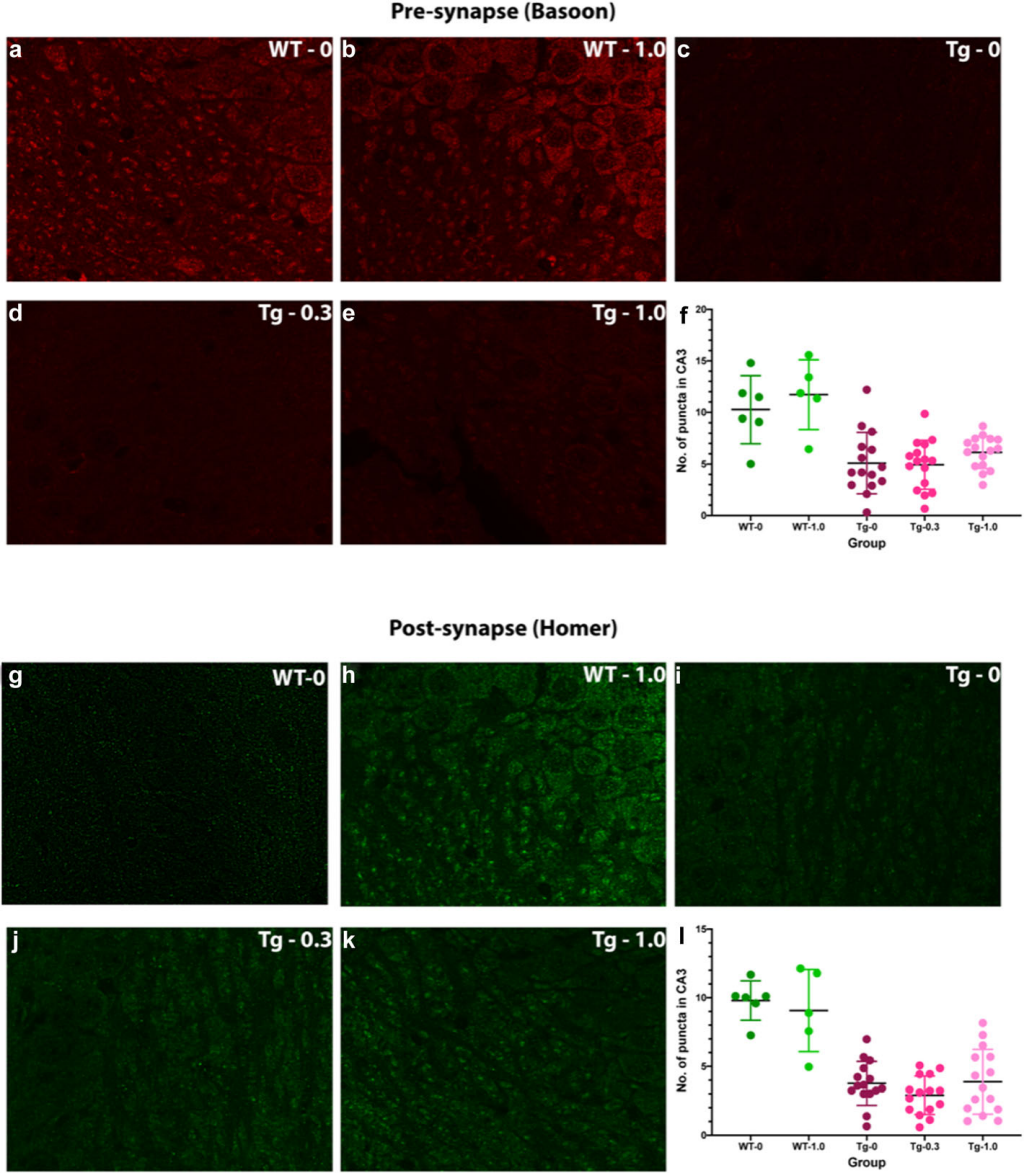
Analysis of pre-synapse and post-synapse in the CA3 region. Brain sections were stained using anti-bassoon (**a**–**e**) or anti-homer (**g**–**k**) antibodies. Representative images are shown for **a**, **g**) wild-type mice treated with vehicle (WT-0), **b**, **h**) wild-type mice treated with 1.0 mg/Kg CLR01 (WT-1.0), **c**, **i**) P301S-tau mice treated with vehicle (Tg-0), **d**, **j**) P301S-tau mice treated with 0.3 mg/Kg CLR01 (Tg-0.3), and **e**, **k**) P301S-tau mice treated with 1.0 mg/Kg CLR01. The data were quantified as the number of puncta per unit area in the CA3 region for Bassoon (**f**) and Homer (**l**)

**Fig. 5:**
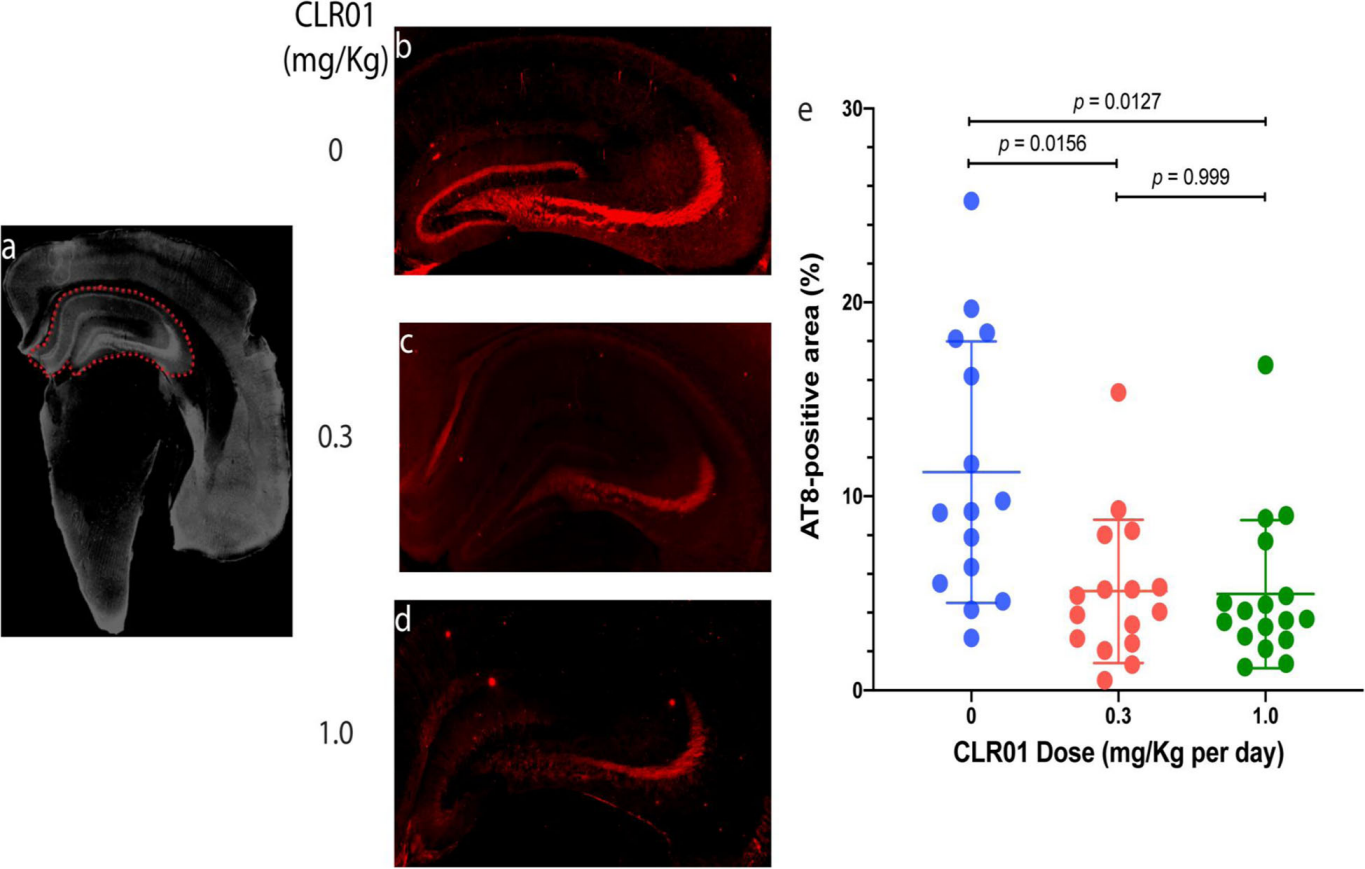
CLR01 treatment reduces hyperphosphorylated tau in the hippocampus of P301S-tau mice. Brain sections from P301S-tau mice were stained with monoclonal antibody AT8 and visualized by immunofluorescence. **a** The typical crescent shape of the hippocampus was delineated by an operator blinded to treatment. **b**–**d** Representative images of the hippocampus area of mice treated with 0 (**a**), 0.3 (**b**), or 1.0 (**c**) mg/kg per day CLR01. **d** The data were quantified as the percentage of AT8-positive area within the hippocampus area, as defined in panel **a**. The data are presented as mean ± SD. P values were calculated using a one-way ANOVA with post hoc Tukey test
