# Supplementary material for: The molecular tweezer CLR01 improves behavioral deficits and reduces tau pathology in P301S-tau transgenic mice
Source: Alzheimers Res Ther. 2021 Jan 4;13:6. doi: 10.1186/s13195-020-00743-x (PMC7784007; doi:10.1186/s13195-020-00743-x)
Supplement: Supplementary file 1 — Additional file 1. [file 13195_2020_743_MOESM1_ESM.docx]

**Supplementary Information**

**The molecular tweezer CLR01 improves behavioral deficits and reduces tau pathology in P301S-tau transgenic mice**

Jing Di^1,§^, Ibrar Siddique^1,§^, Zizheng Li^1^, Ghattas Malki^1^, Simon Hornung^1†^, Suman Dutta^1^, Ian Hurst^1^, Ella Ishaaya^1^, Austin Wang^1^, Sally Tu^1^, Ani Boghos^1^, Ida Ericsson^1^,Frank-Gerrit Klärner^4^, Thomas Schrader^4^, and Gal Bitan^1,2,3,^*

^1^Department of Neurology, David Geffen School of Medicine at UCLA, ^2^Brain Research Institute, and ^3^Molecular Biology Institute, University of California, Los Angeles, CA USA. ^4^Faculty of Chemistry, University of Duisburg-Essen, Germany.


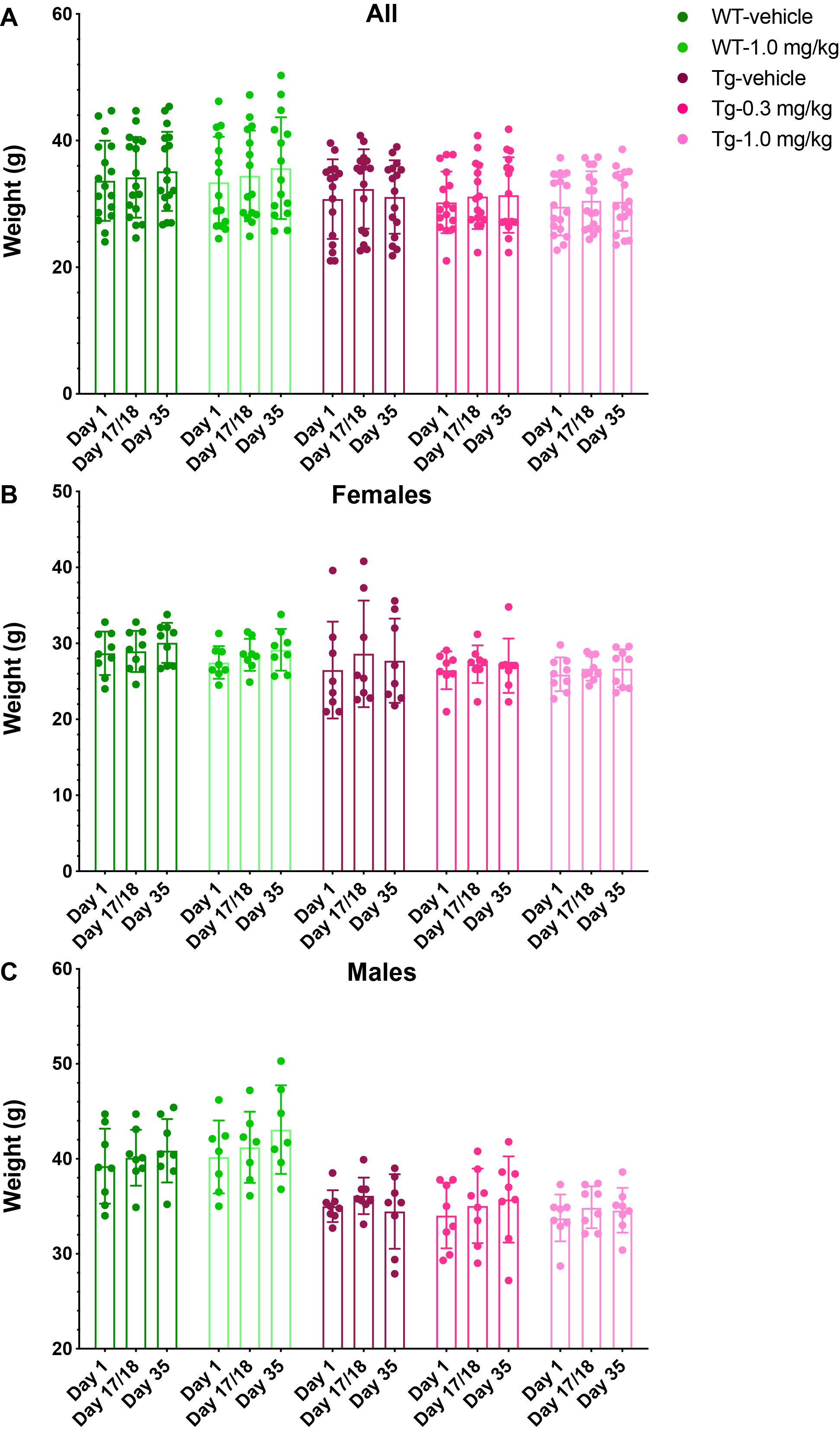


**Supplementary Figure S1. Mouse weight change during the treatment.** Mice were weighed on days 1, 17/18, and 35 of the treatment. The data are presented for A) all the mice in each group, B) female mice, and C) male mice as mean ± SD.


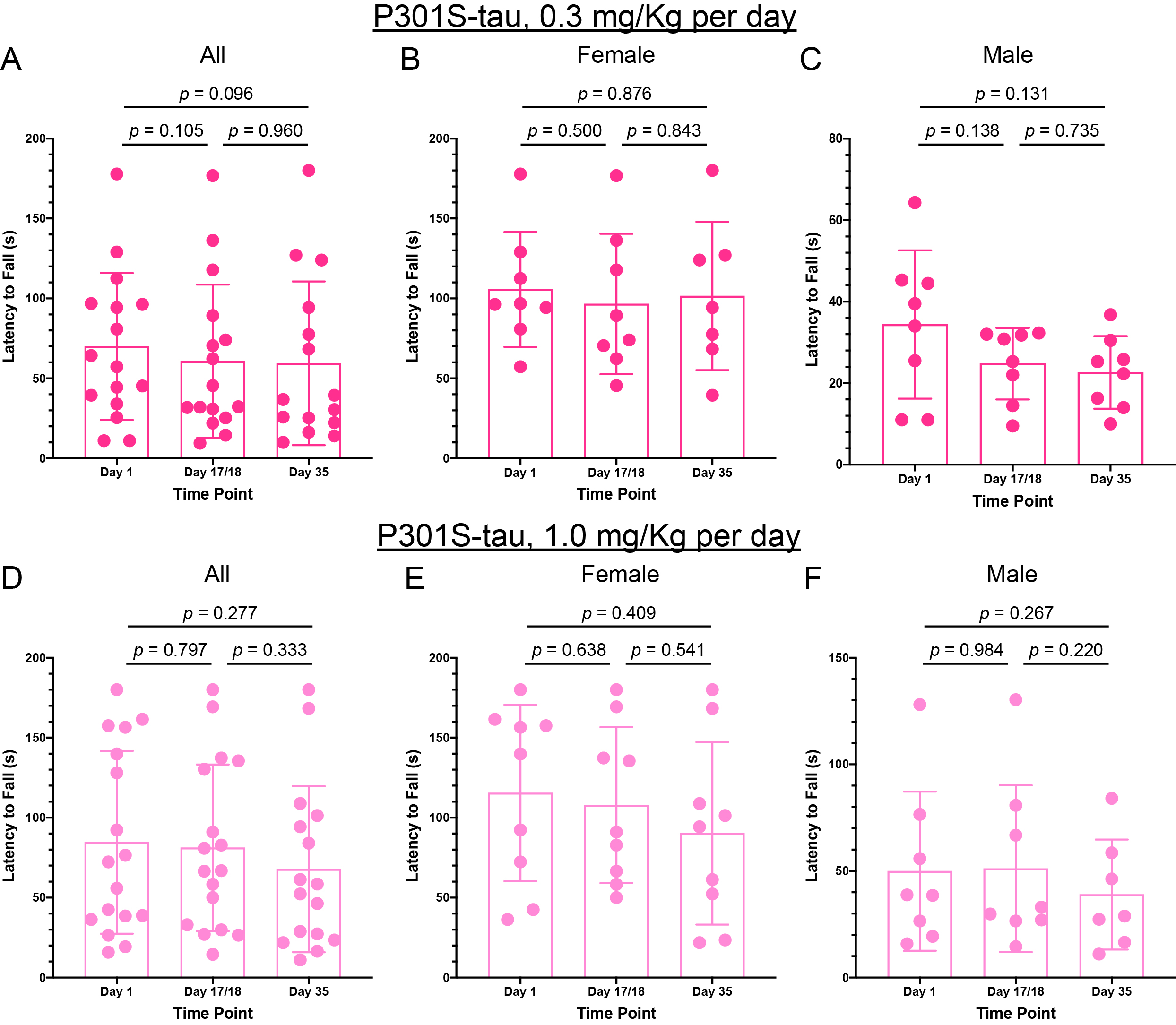


**Supplementary Figure S2. Grip-strength does not change significantly in CLR01-treated P301S-tau mice.** P301S-tau mice were tested using the grip-strength assay on days 1, 17/18, and 35 of the treatment. A–C) Treatment effect in all (A), female (B), and male (C) P301S-tau mice in the low-dose group. D–F) Treatment effect in all (D), female (E), and male (F) P301S-tau mice in the high-dose group. The data are presented as mean ± SD. P-values were calculated using a one-way, repeated-measure ANOVA with *post hoc* Tukey test.


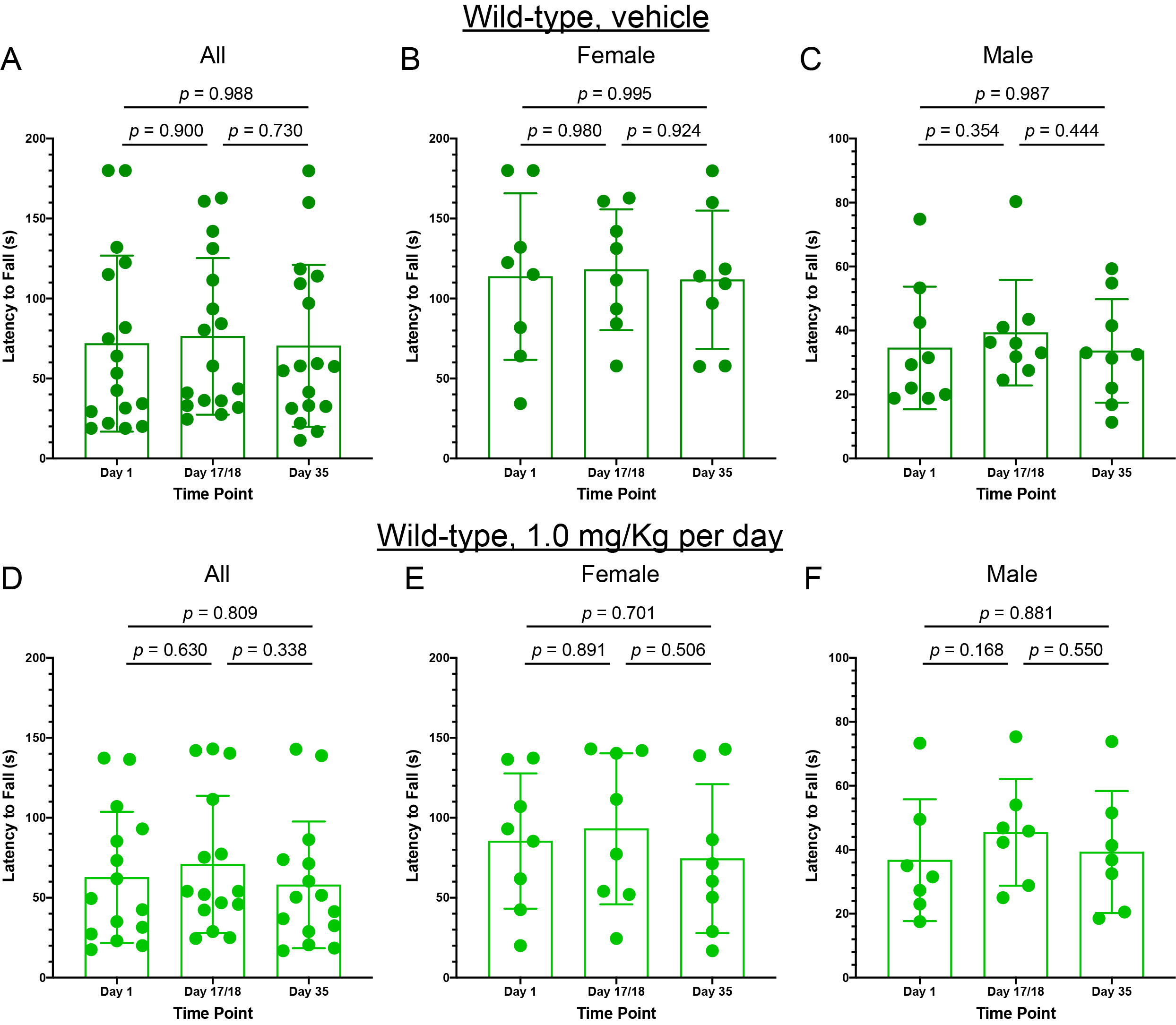


**Supplementary Figure S3. Grip-strength does not change significantly in wild-type mice.** Wild-type littermates of the P301S-tau mice used in the study were subjected to the grip-strength test on days 1, 17/18, and 35 of the treatment. A–C) Grip-strength in all (A), female (B), and male (C) vehicle-treated wild-type mice. D–F) Treatment effect in all (D), female (E), and male (F) wild-type mice receiving 1.0 mg/Kg per day CLR01. The data are presented as mean ± SD. P-values were calculated using a one-way, repeated-measure ANOVA with *post hoc* Tukey test.


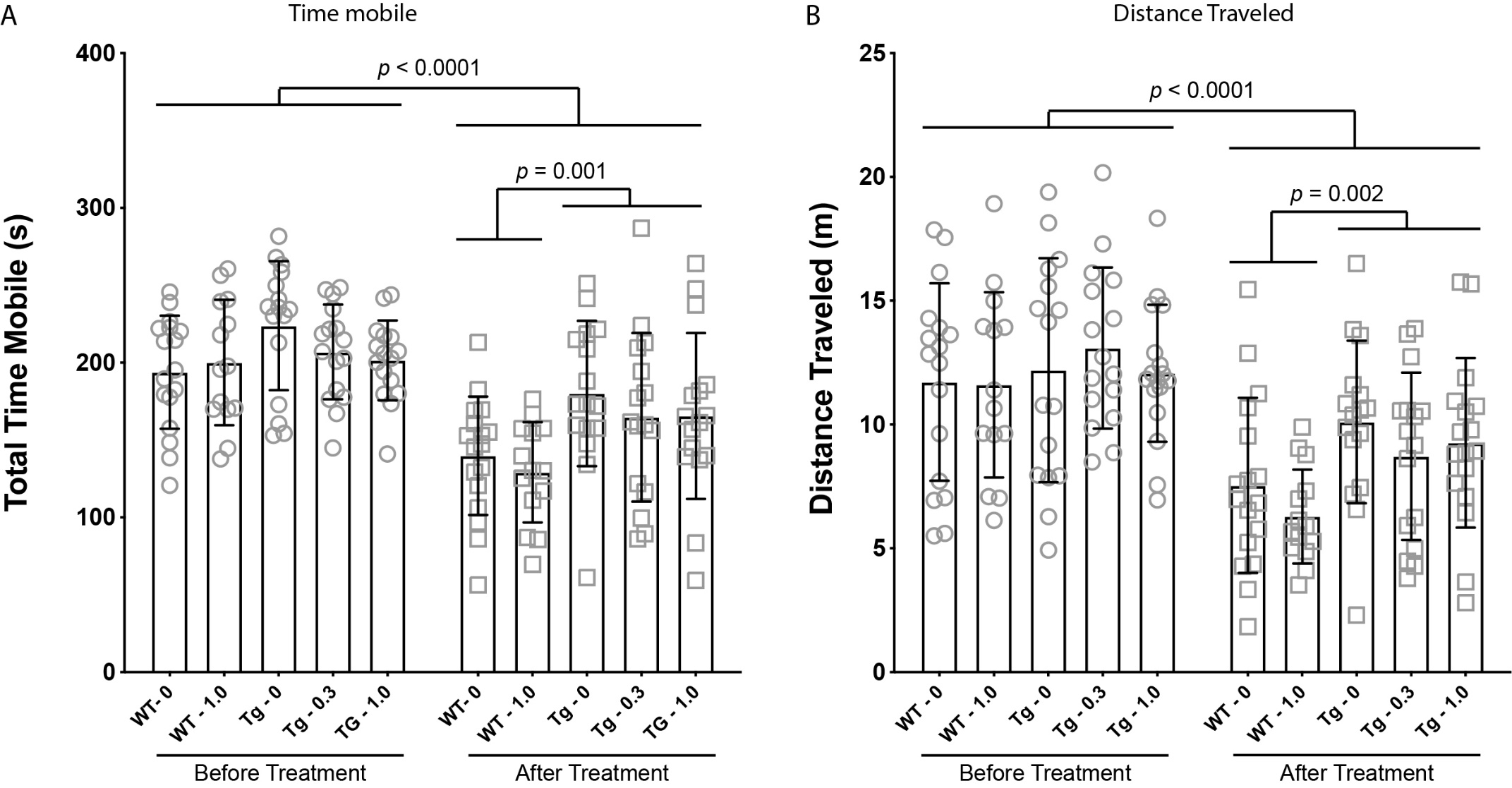


**Supplementary Figure S4. Differences in open-field behavior between P301S-tau and wild-type mice before and after treatment.** All the mice were examined in the beginning and end of the treatment for 5 min in the open-field test. All sessions were video-recorded and data were analyzed using ANY-maze. A) Total time (in seconds) each individual animal was mobile during the test. B) Total distance travelled (in meters) by each individual animal during the test. Data are presented as mean ± SD. P-values were calculated first using a mixed-effect, repeated-measure model with *post hoc* Sidak test (not shown). Then, the grouped p-values shown in the figure were calculated using unpaired t-tests.


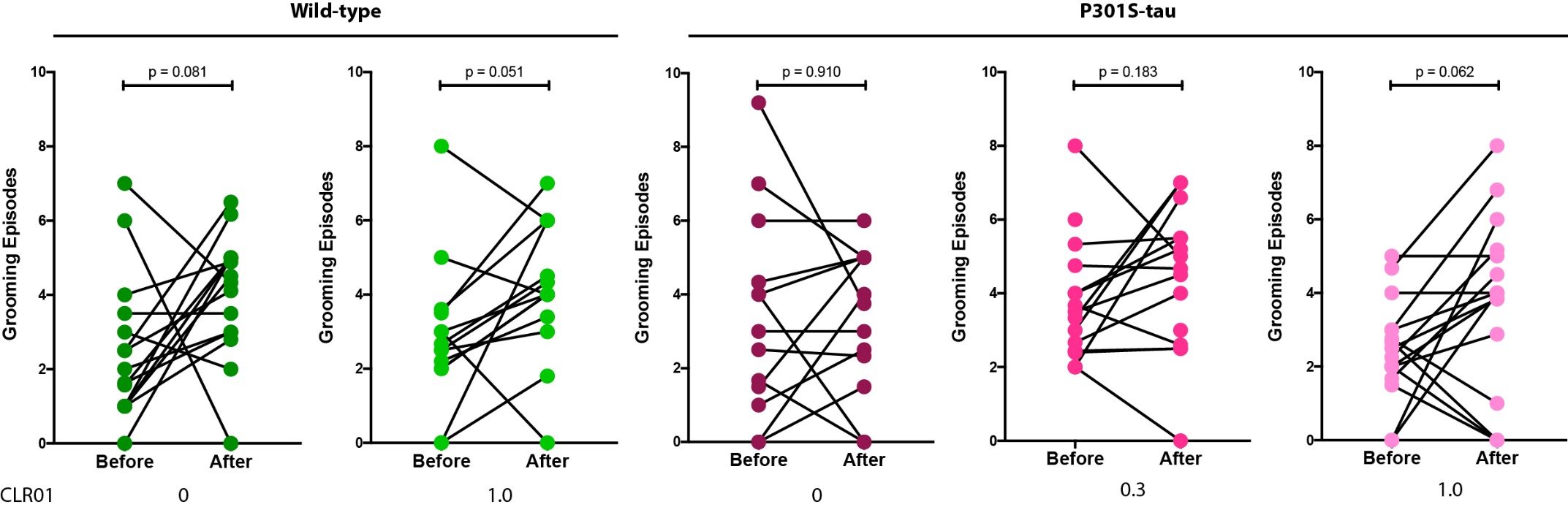


**Supplementary Figure S5. CLR01 treatment moderately increases P301S-tau mice grooming.** The number of grooming episodes before (day 1) and after (day 35) the treatment was recorded manually by a blinded observer using the video recordings of the open-field test. P-values for the differences between the two time points in each group were calculated using paired t-tests.


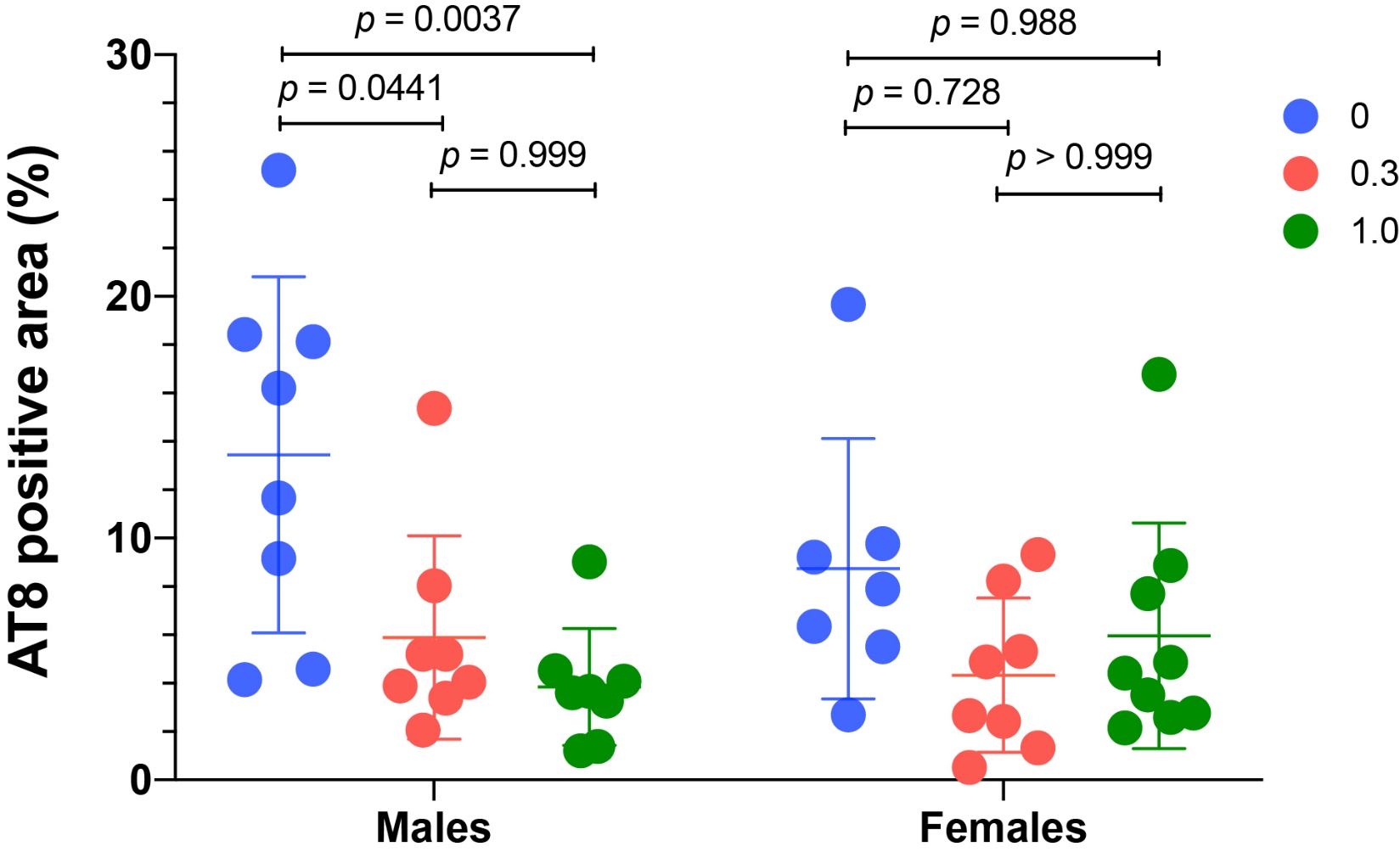


**Supplementary Figure S6. The decrease in hyperphosphorylated tau in the hippocampus of P301S-tau mice is mainly driven by the males.** Brain sections from P301S-tau mice were stained with monoclonal antibody AT8 and visualized by immunofluorescence. The data were quantified as the percentage of AT8-positive area in the hippocampus and are presented as mean ± SD. P-values were calculated using a two-way ANOVA with *post hoc* Tukey test.


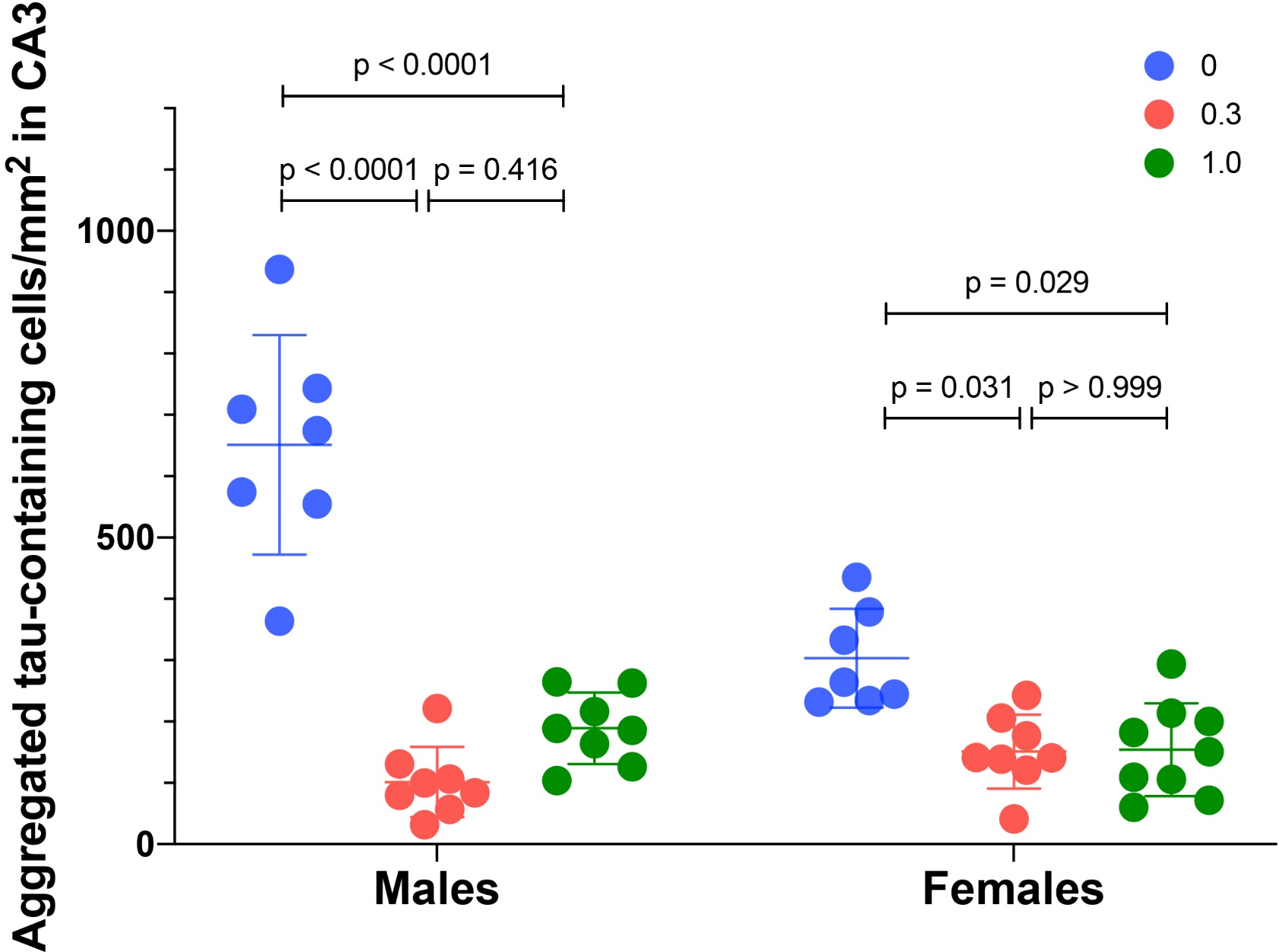


**Supplementary Figure S7. Analysis by sex of CLR01’s effect on tau aggregation in the CA3 region of P301S-tau mice.** Brain sections from P301S-tau mice were stained with Gallyas silver-stain. The data were quantified as the number of tau-aggregate-containing cells per mm^2^ in the CA3 and are presented as mean ± SD. P-values were calculated using a two-way ANOVA with *post hoc* Sidak test.


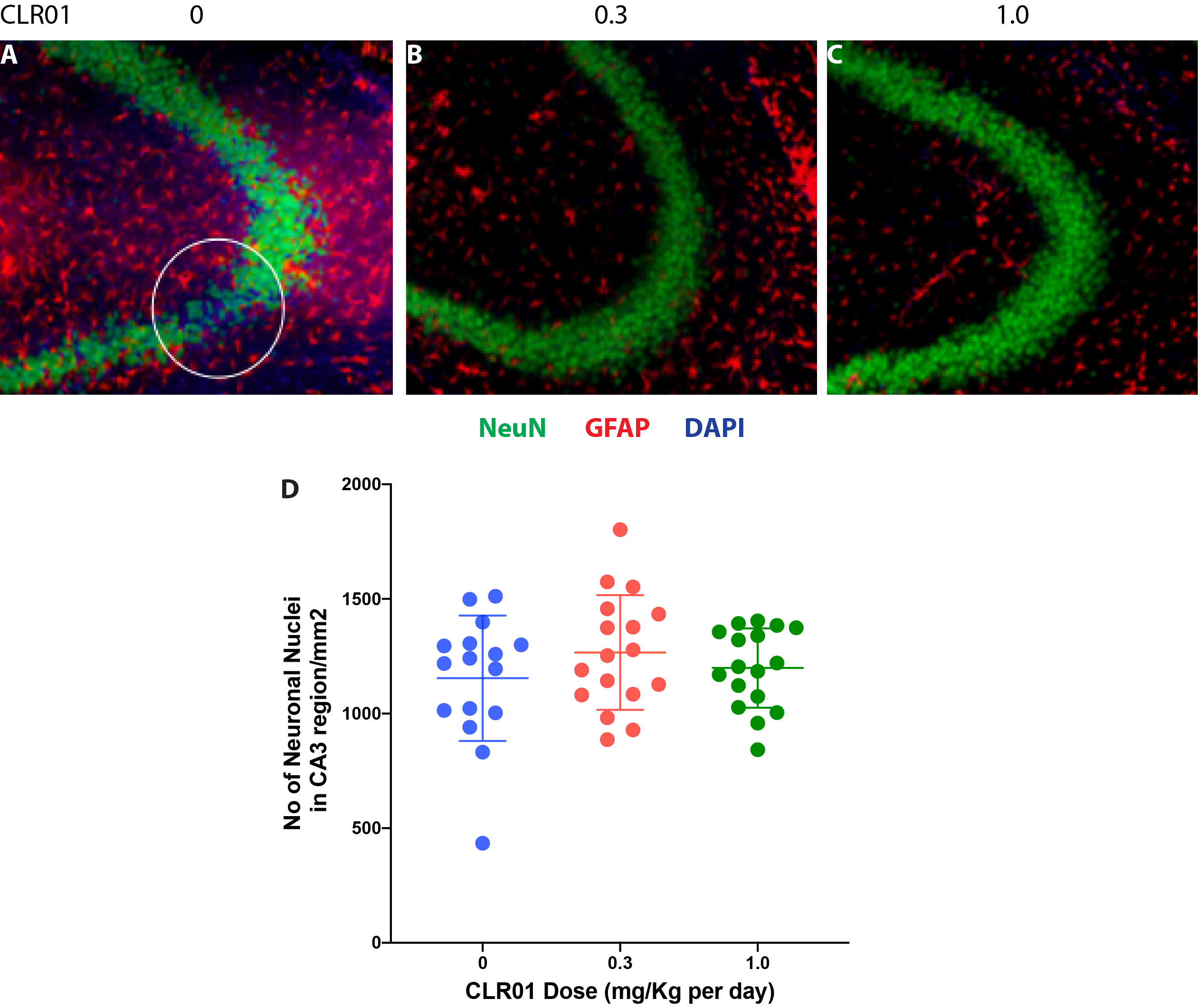


**Supplementary Figure S8. Analysis of neurodegeneration in the CA3 region of P301S-tau mice.** Brain sections from P301S-tau mice were stained using an anti-NeuN antibody (green) and counter-stained using an anti-GFAP antibody (red), to facilitate delineation of the CA3 region, and DAPI (blue). Representative images are shown for individual mice from the A) vehicle-treated, B) low-dose, and C) high-dose groups. The circle in panel A shows an area of neurodegeneration observed in two out of the eight mice in this group. D) The data were quantified as the number of neuronal nuclei per mm^2^ in the CA3 and are presented as mean ± SD. P-values were calculated using a two-way ANOVA with *post hoc* Tukey test.


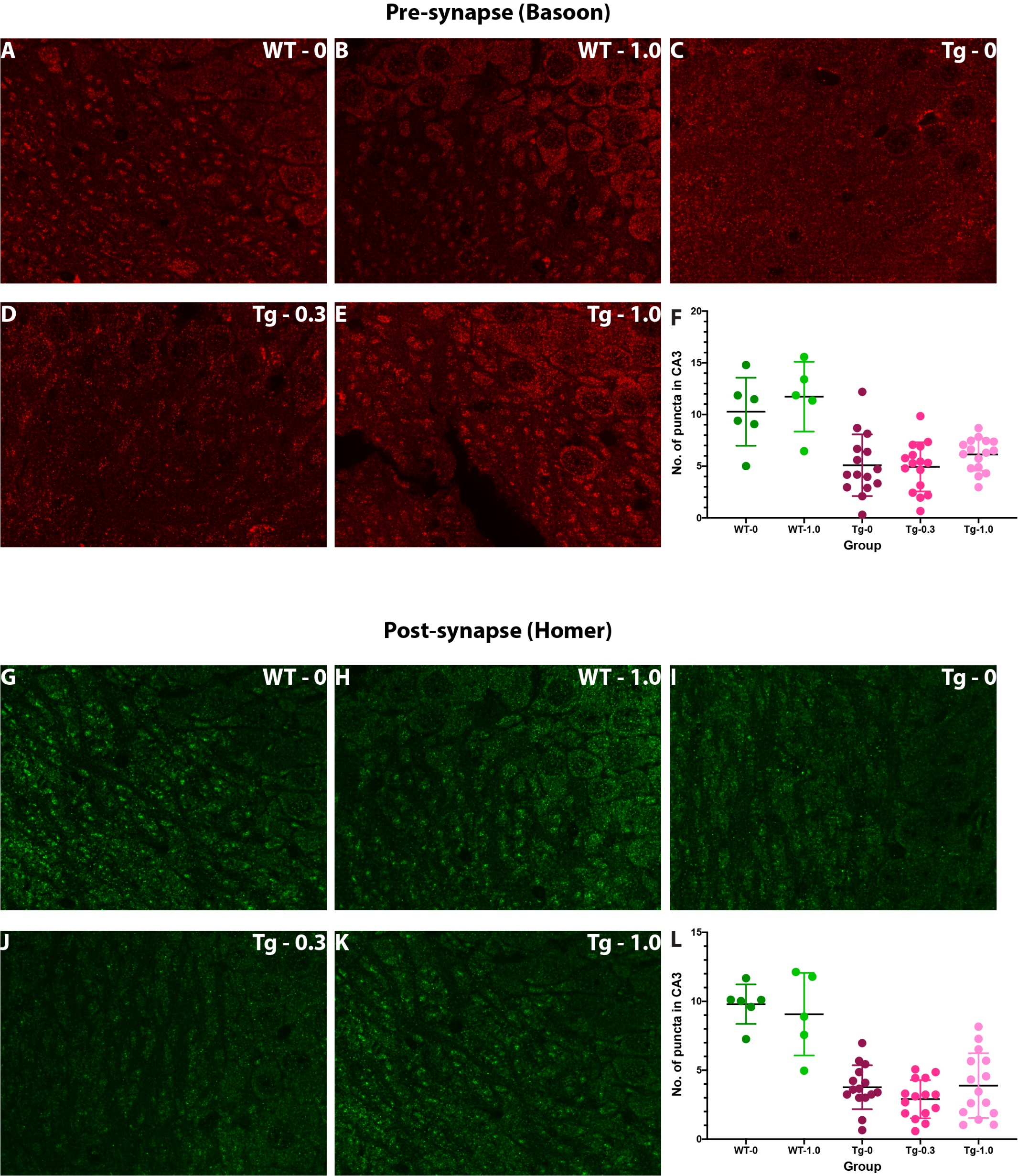


**Supplementary Figure S9. Analysis of pre-synapse and post-synapse in the CA3 region.** Brain sections were stained using anti-bassoon (A–E) or anti-homer (G–K) antibodies. Representative images are shown for A,G) wild-type mice treated with vehicle (WT-0), B,H) wild-type mice treated with 1.0 mg/Kg CLR01 (WT-1.0), C,I) P301S-tau mice treated with vehicle (Tg-0), D,J) P301S-tau mice treated with 0.3 mg/Kg CLR01 (Tg-0.3 ), and E,K) P301S-tau mice treated with 1.0 mg/Kg CLR01. The data were quantified as the number of puncta per unit area in the CA3 region for Bassoon (F) and Homer (L).


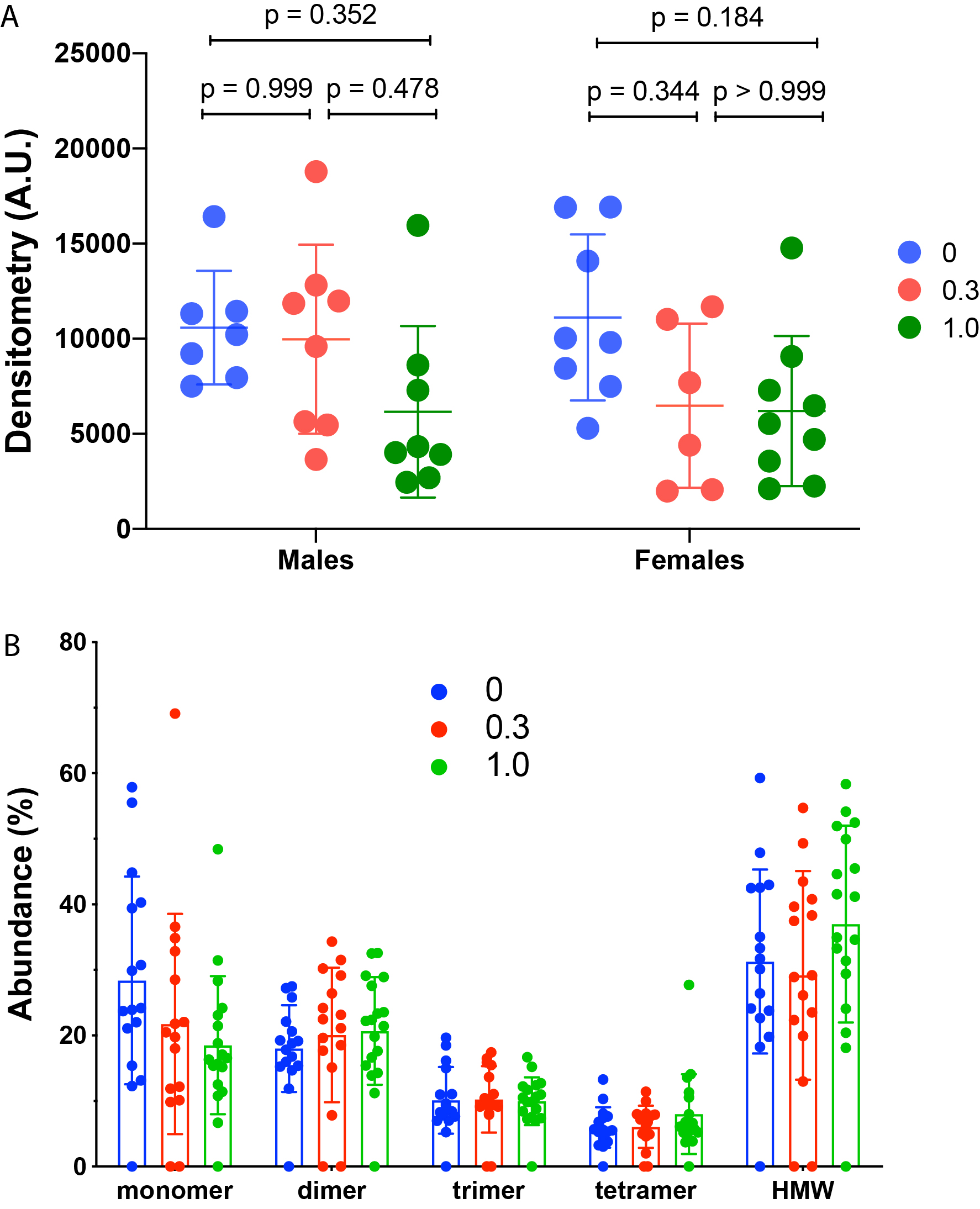


**Supplementary Figure S10. Analysis of tau oligomerization in P301S-tau mice.** The soluble fraction of the hippocampus extract of P301S-tau mice was examined by A) dot blots probed using the anti-tau-oligomer antibody TOC-1, or B) native-PAGE/western blot probed with monoclonal antibody HT7. The dot-blots were quantified by densitometry. The data are presented as mean ± SD. P-values were calculated using a two-way ANOVA with *post hoc* Tukey test. The p-values in panel B were ≥ 0.107 and are not shown in the figure.


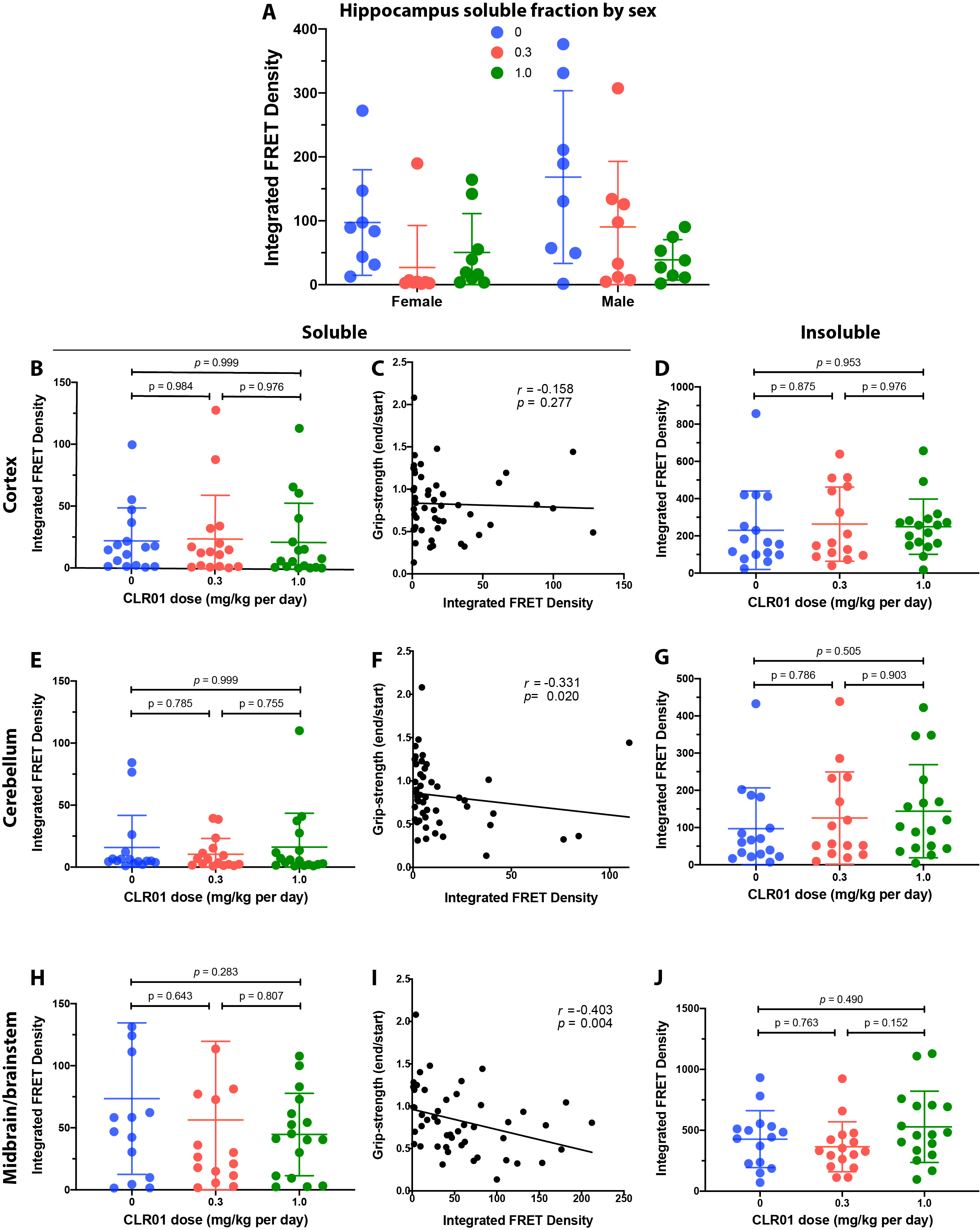


**Supplementary Figure S11. CLR01’s effect on tau seeds in extracts from different brain regions in tau-biosensor cells.** The soluble and insoluble fraction of brain extracts from P301S-tau mice were added to tau-biosensor cells and incubated for 48 h. Integrated FRET density was measured by flow cytometry. A) Hippocampus soluble fraction analyzed separately for female and male mice. The data are presented as mean ± SD. P-values were calculated using a two-way ANOVA with *post hoc* Tukey test. B–G) similar analyses of the soluble (B, D, F) and insoluble (C, E, G) fractions from extracts of the cortex (B, C), cerebellum (D, E), or midbrain and brainstem (F, G). P-values were calculated using a one-way ANOVA with *post hoc* Tukey test.


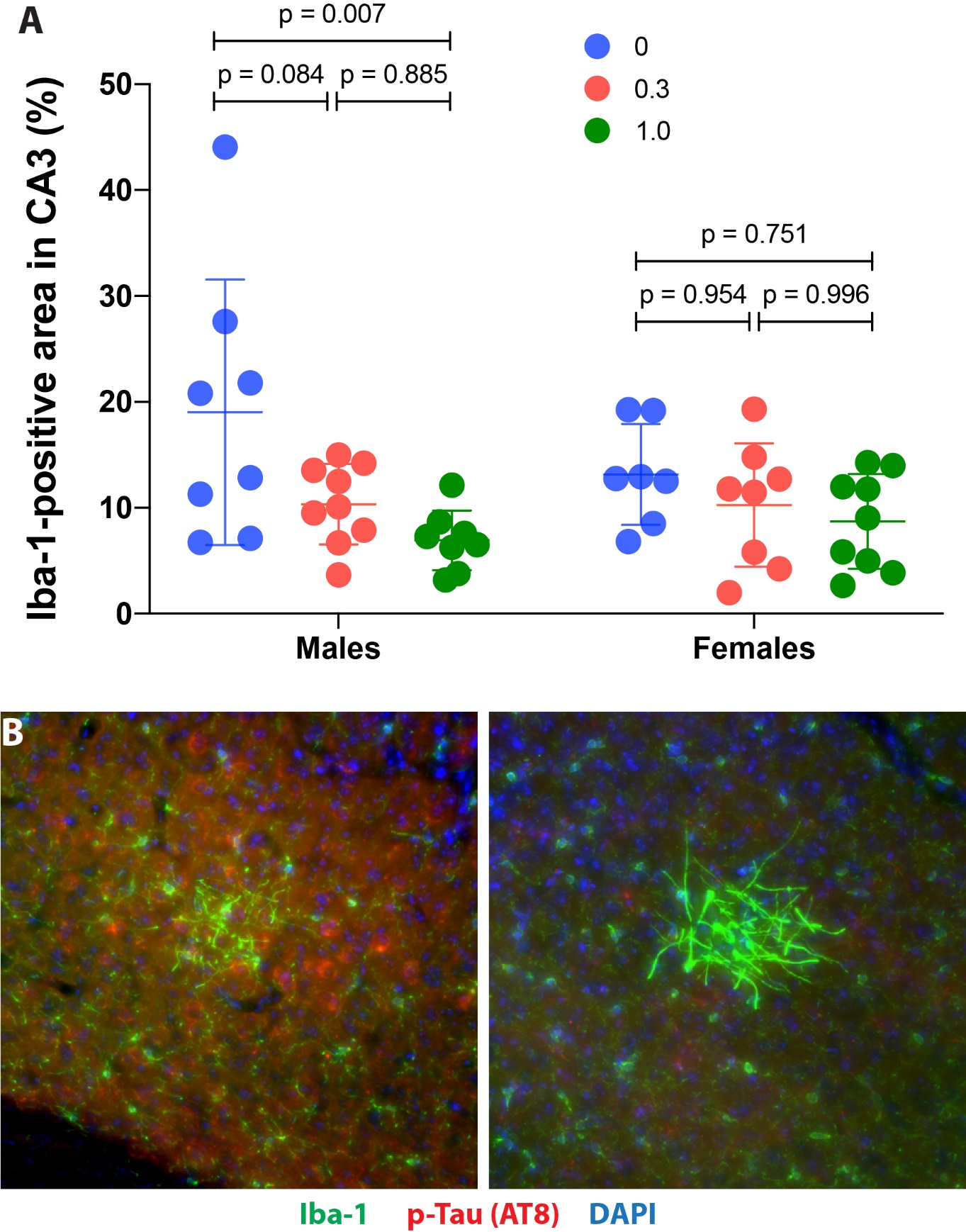


**Supplementary Figure S12. Analysis of microglia in P301S-tau mice.** A) Brain sections from P301S-tau mice were stained with an anti-Iba-1 antibody and visualized by immunofluorescence. The data were quantified as the percentage of Iba-1-positive area in the CA3. The data are presented as mean ± SD. P-values were calculated using a two-way ANOVA with *post hoc* Tukey test. B) Peculiar, rare clusters of Iba-1 staining in the cortex of two female mice in the vehicle-treated P301S-tau mice. Similar clusters were not found in other mice or other brain regions.


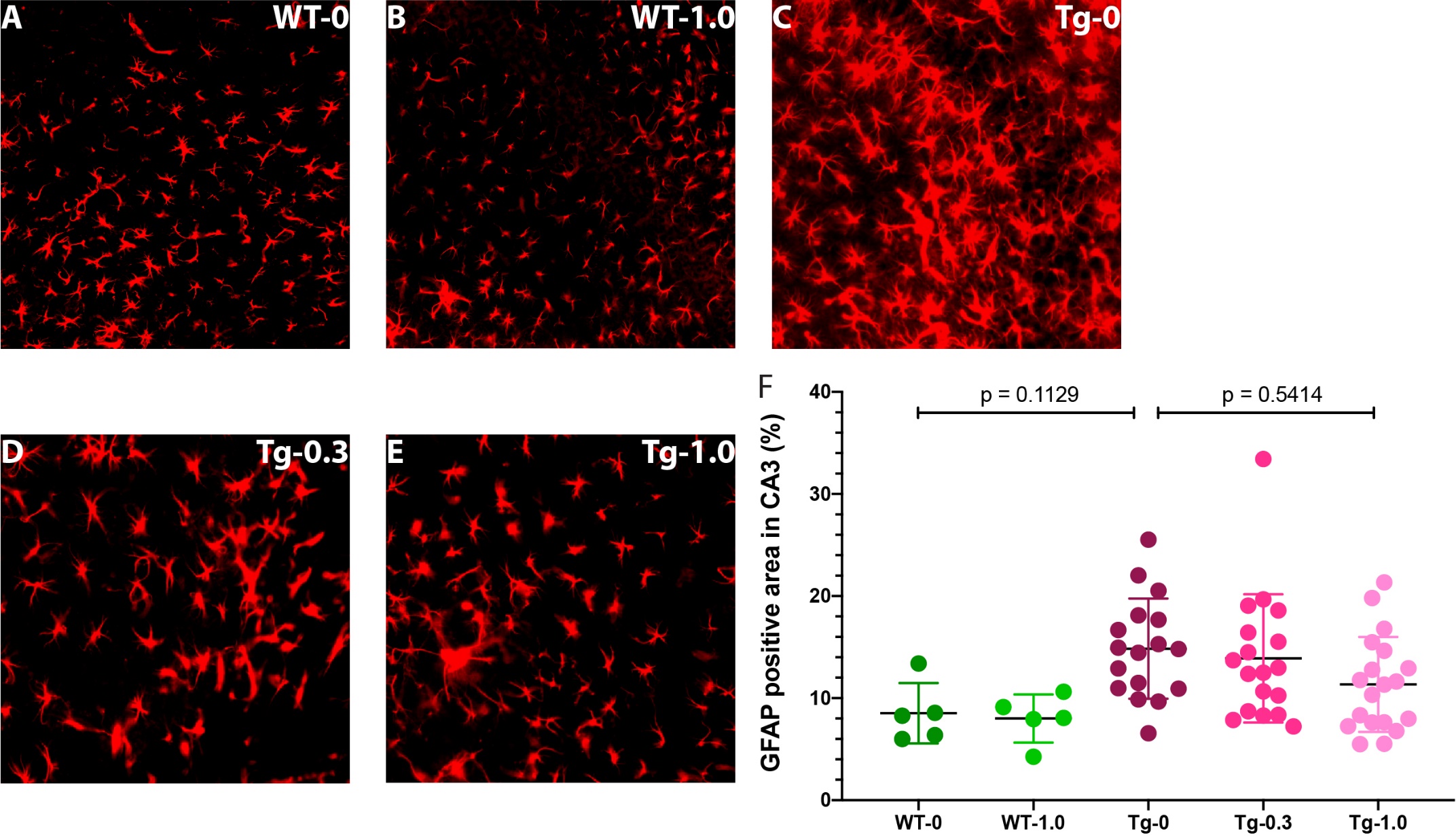


**Supplementary Figure S13. CLR01 treatment shows a trend toward reduction of GFAP staining in the CA3 region of P301S-tau mice.** Brain sections from P301S-tau mice and wild-type littermates were stained using an anti-GFAP antibody and visualized by immunofluorescence. A-E) Representative images of the CA3 area of wild-type (WT) mice treated with 0 (A) or 1.0 (B) mg/Kg CLR01 and transgenic (Tg) P301S-tau mice treated with 0 (C), 0.3 (D), or 1.0 (E) mg/Kg CLR01. F) The data were quantified as the percentage of GFAP-positive area in the CA3 and are presented as mean ± SD. P-values were calculated using a one-way ANOVA with *post hoc* Tukey test.
